# Moving Beyond the Host: Unraveling the Skin Microbiome of Endangered Costa Rican Amphibians

**DOI:** 10.3389/fmicb.2019.02060

**Published:** 2019-09-12

**Authors:** Randall R. Jiménez, Gilbert Alvarado, Josimar Estrella, Simone Sommer

**Affiliations:** ^1^Institute of Evolutionary Ecology and Conservation Genomics, University of Ulm, Ulm, Germany; ^2^Laboratory of Comparative Wildlife Pathology, School of Veterinary Medicine and Animal Sciences, University of São Paulo, São Paulo, Brazil; ^3^Laboratory of Experimental and Comparative Pathology (LAPECOM), Biology School, University of Costa Rica, San José, Costa Rica

**Keywords:** skin bacterial diversity, 16S rRNA amplicon sequencing, *Lithobates vibicarius*, *Craugastor escoces*, *Isthmohyla rivularis*, *Isthmohyla pseudopuma*, *Batrachochytrium dendrobatidis*, health implications

## Abstract

Some neotropical amphibians, including a few species in Costa Rica, were presumed to be “extinct” after dramatic population declines in the late 1980s but have been rediscovered in isolated populations. Such populations seem to have evolved a resistance/tolerance to *Batrachochytrium dendrobatidis* (*Bd*), a fungal pathogen that causes a deadly skin disease and is considered one of the main drivers of worldwide amphibian declines. The skin microbiome is an important component of the host’s innate immune system and is associated with *Bd*-resistance. However, the way that the bacterial diversity of the skin microbiome confers protection against *Bd* in surviving species remains unclear. We studied variation in the skin microbiome and the prevalence of putatively anti-*Bd* bacterial taxa in four co-habiting species in the highlands of the Juan Castro Blanco National Park in Costa Rica using 16S rRNA amplicon sequencing. *Lithobates vibicarius*, *Craugastor escoces*, and *Isthmohyla rivularis* have recently been rediscovered, whereas *Isthmohyla pseudopuma* has suffered population fluctuations but has never disappeared. To investigate the life stage at which the protective skin microbiome is shaped and when shifts occur in the diversity of putatively anti-*Bd* bacteria, we studied the skin microbiome of tadpoles, juveniles and adults of *L. vibicarius*. We show that the skin bacterial composition of sympatric species and hosts with distinct *Bd*-infection statuses differs at the phyla, family, and genus level. We detected 94 amplicon sequence variants (ASVs) with putative anti-*Bd* activity pertaining to distinct bacterial taxa, e.g., *Pseudomonas* spp., *Acinetobacter johnsonii*, and *Stenotrophomonas maltophilia*. *Bd-*uninfected *L. vibicarius* harbored 79% more putatively anti-*Bd* ASVs than *Bd-*infected individuals. Although microbiome composition and structure differed across life stages, the diversity of putative anti-*Bd* bacteria was similar between pre- and post-metamorphic stages of *L. vibicarius*. Despite low sample size, our results support the idea that the skin microbiome is dynamic and protects against ongoing *Bd* presence in endangered species persisting after their presumed extinction. Our study serves as a baseline to understand the microbial patterns in species of high conservation value. Identification of microbial signatures linked to variation in disease susceptibility might, therefore, inform mitigation strategies for combating the global decline of amphibians.

## Introduction

All vertebrates harbor a diverse and dynamic microbial community on their skin – the so-called skin microbiome – with which they have co-evolved (McFall-Ngai et al., 2013; Pita et al., 2018). The skin microbiome is considered an important component of the host’s innate immune system, being one of the first lines of defense against pathogenic infections and the mediator of disease susceptibility (Harris et al., 2006; King et al., 2016). In vertebrates, adequate protection against pathogens has been linked with distinct characteristics of the microbiome, such as bacterial species richness, microbial community assemblage, and the presence of bacteria that produce certain antimicrobial compounds that inhibit pathogen growth (Harris et al., 2006; Lemieux-Labonté et al., 2017; Ellison et al., 2018).

In amphibians, the skin microbiome can vary across species and populations, being shaped, for example, by ontogeny, geography, and seasonality (Kueneman et al., 2014; Longo et al., 2015; Bletz et al., 2017; Prest et al., 2018). Notably, it can protect against the infection of the fungal pathogen *Batrachochytrium dendrobatidis* (hereafter *Bd*), which causes an infectious skin-disease called chytridiomycosis that can kill amphibians through the production of noxious compounds and disruption of cutaneous osmoregulation (Berger et al., 1998; Voyles et al., 2009). In particular, skin bacteria can inhibit *Bd* colonization and pathogenicity in amphibians (Harris et al., 2009; Muletz-Wolz et al., 2017a). Previous research has shown that the presence of *Bd*-inhibitory bacteria (e.g., *Acinetobacter*, *Lysobacter*, *Janthinobacterium*) provides a protective function against *Bd* in certain hosts (Brucker et al., 2008; Woodhams et al., 2018; Rebollar et al., 2019). Characterization of these microbiome patterns across hosts should guide us to a better understanding of co-evolutionary processes and may provide means to protect amphibians against pathogens such as *Bd*.

Amphibians, especially those in the Neotropics, have experienced severe population declines and extinctions since the 1980s (Whitfield et al., 2007, 2016). In Costa Rica, one of the most amphibian-diverse countries in the world, numerous amphibian populations from the highlands drastically declined during the 1980s and 1990s, to the extent of the possible extirpation of entire species (Bolaños, 2009). A well-known example is the enigmatic extinction of the endemic golden toad (*Incilius periglenes*) from the protected Monteverde cloud forest (Crump et al., 1992; Pounds et al., 1997). This horrifying situation has been mainly attributed to outbreaks of *Bd*-disease together with factors such as habitat alteration, environmental contaminants, and climate change (Collins and Storfer, 2003; Skerratt et al., 2007; Whitfield et al., 2016). Molecular evidence suggests that *Bd* was present in Costa Rica prior to the observed declines, and distribution models have shown that *Bd* has spread around the country to the detriment of many amphibian populations (Puschendorf et al., 2006, 2009; De León et al., 2018).

Some species that suffered sharp reductions in the abundance of their populations have since shown signs of recovery. Others that were presumed extinct after the extirpation of their known populations, surprisingly, have been rediscovered years later in small and isolated populations (so-called “relict populations”), despite *Bd* still being present (Pounds et al., 1997; González-Maya et al., 2013; Chaves et al., 2014; Whitfield et al., 2017). Recovering and relict populations seem to have evolved resistance/tolerance to *Bd*. A protective skin microbiome has been suggested to be one of the most important factors providing this resistance to *Bd* (Woodhams et al., 2007b, 2016; Grogan et al., 2018). Among these resistant populations are four sympatric amphibian species from the highlands of Costa Rica above 1000 m.a.s.l. Three of them, namely *Lithobates vibicarius*, *Craugastor escoces*, and *Isthmohyla rivularis*, persist despite *Bd* presence after their presumed disappearance/extinction. *L. vibicarius* declined drastically and apparently disappeared across its range between the late 1980s and mid-1990s, but surprisingly, since 2002, relict populations have been re-encountered in Costa Rica at a few sites coexisting with *Bd* (Castro-Cruz and García-Fernández, 2012; Whitfield et al., 2016) with relatively stable numbers of adults. *C. escoces*, which was once abundant across its distribution range, was declared “Extinct” by the IUCN, but a single individual was recently found by our research group after 30 years without sightings and 12 years of being declared extinct (Jiménez and Alvarado, 2017). It is noteworthy that its last detection had preceded the last sighting of the famous golden toad, *I. periglenes.* Two years of continuous surveys following the rediscovery of the single female have revealed only one new adult (Douglas Vargas, personal communication). The frog *I. rivularis* also declined in the late 1980s, but since 2007 has occasionally been reported in distinct locations, such as the Monteverde cloud forest (Solís et al., 2010) and our study area, the Juan Castro Blanco National Park (Jiménez et al., 2019). The other species, *Isthmohyla pseudopuma*, exists with apparently stable populations, although fluctuations in abundance were observed before and after the declines in the populations from Monteverde (Pounds et al., 1997). These *Bd*-survivor species, particularly those that were presumed extinct, provide a unique opportunity to improve our understanding of the selection and diversity of *Bd*-protective microbiomes in neotropical amphibians.

In the present study, we examined the four above-mentioned sympatric frog species to describe the variation in their skin microbiomes and investigate the prevalence of putatively anti-*Bd* bacterial members according to *Bd-*infection status. Previous research has shown that the amphibian skin microbiome is species-specific because of the traits with which it associates, such as skin compounds (e.g., antimicrobial peptides), ecology, and behavior (McKenzie et al., 2011; Kueneman et al., 2014; Belden et al., 2015). Therefore, we hypothesized that skin bacterial communities are distinct among adults of different study species. Furthermore, recent evidence has shown distinct microbiome diversity between *Bd* infected/uninfected amphibians (Ellison et al., 2018), as well as direct interactions between *Bd* and skin microbes, whereby some bacterial taxa can inhibit *Bd* growth, colonization, and establishment on the skin of certain hosts (Harris et al., 2009; Park et al., 2014; Becker et al., 2015; Muletz-Wolz et al., 2017a, b). Consequently, if the skin microbiome plays a significant role in *Bd*-resistance, we would expect a different microbiome between *Bd*-infected and *Bd*-uninfected adults, and *Bd*-uninfected individuals should harbor a higher diversity of putatively anti-*Bd* bacterial members.

In order to determine the life stage at which the protective skin microbiome is shaped, as well as when shifts occur in the abundance of putatively anti-*Bd* bacteria, we focused on *L. vibicarius*. It is well known that dramatic changes occur during amphibian metamorphosis, such as the keratinization of skin tissue and the development of the immune system (Wells, 2007; Rollins-Smith, 2009). Keratinized tissue in amphibians is restricted to the mouthparts of tadpoles and the skin of juveniles and adults (Alibardi, 2001; Wells, 2007). The mucus glands present before and after metamorphosis produce distinct secretions that act as a physical protective barrier and substrate for skin microbial communities, which limit and/or promote the growth of certain bacterial taxa (Rollins-Smith, 2009; Woodhams et al., 2014). *Bd* infects both larval and post-metamorphic stages but it usually does not produce lethal infections until after metamorphosis, when skin becomes keratinized (Blaustein et al., 2005; Langhammer et al., 2014). Thus, we hypothesized that the skin microbiome differs between the life stages of *L. vibicarius*. Given that juveniles and adults are usually more susceptible to *Bd* than tadpoles (Blaustein et al., 2005; Langhammer et al., 2014) – presumably due to increased keratin distribution and abundance after the larval stage – we expected an increase in the diversity of putatively anti-*Bd* bacteria after metamorphosis in *L. vibicarius*.

## Materials and Methods

### Study Sites

We surveyed four study sites in the Juan Castro Blanco National Park and adjacent private lands within Alajuela province, Costa Rica (10° 16′ 44.4^°^ N, 84° 19′ 58.9^°^ W) for the presence of amphibians over three consecutive years [2015 (year 1), 2016 (year 2), and 2017 (year 3)] during the same time period within the rainy season (September–November). We have not provided the exact coordinates of the study sites to discourage people from visiting them, because we hope to prevent the potential introduction of pathogens and illegal collecting.

Three sites (Lagunillas, Monjes, and Congo) are disturbed landscapes adjacent to the national park. Each of these sites contains a pond and small streams within pastures for cattle and are surrounded by secondary and fragmented montane humid forest. The presence of cow feces, the antibiotic treatment of cows, and the use of pesticides (e.g., herbicides) at these sites might negatively affect habitat quality for local amphibians. The national park site (hereafter NP) includes three closely located large ponds surrounded by late successional and continuous montane humid forest. The altitudes of the study sites range from 1830 to 1900 m.a.s.l.

All four study sites have been monitored since 2015 for the presence of *Bd* by our research group (see below). *L. vibicarius* ceased to occur at these sites in the late 1980s (Douglas Vargas, personal communication), coinciding with the amphibian population declines and extirpations thought to be associated with *Bd* during the late 1990s.

### Focal Species

**FIGURE 1:**
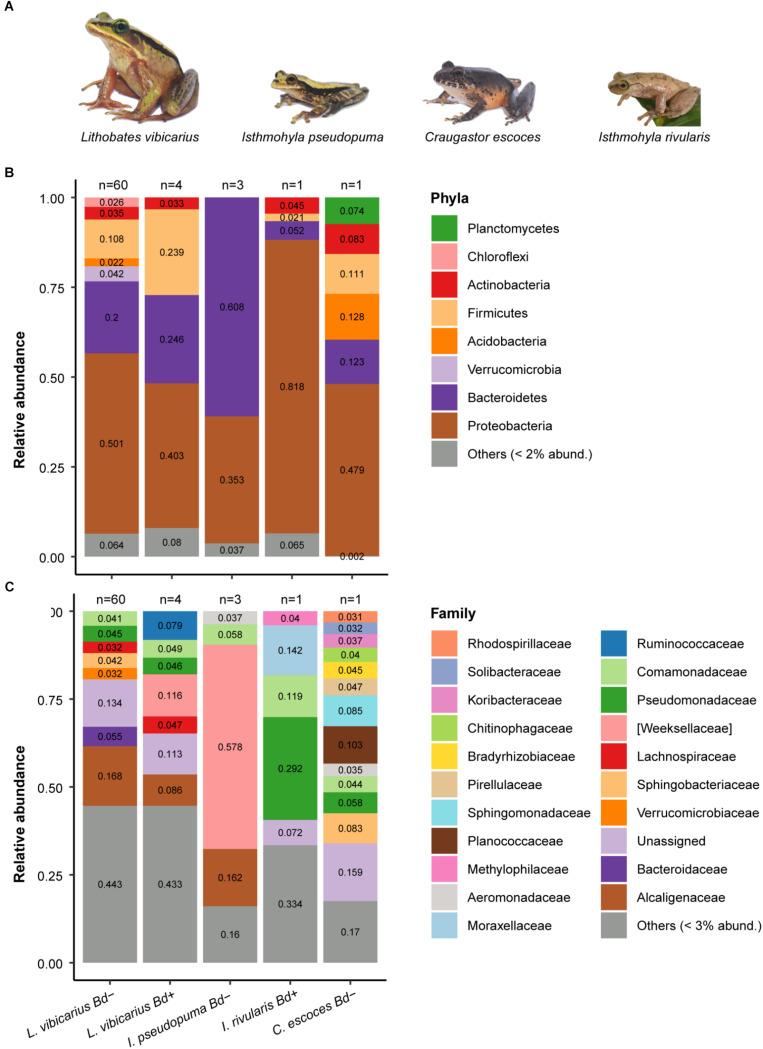
Skin microbiome of four adult frog species grouped by *Bd*-infection status. **(A)** Neotropical montane frog species used for studying the host-associated skin microbiome **(B)** Relative abundance of skin bacterial taxa at the phylum level across species. Rare ASVs (relative abundances <2%) were clustered together. **(C)** Relative abundance of skin bacterial taxa at the family level across species. ASVs with relative abundances <3% were clustered together.

We detected four amphibian species in study sites (Figure 1A). The most abundant species, *L*, *vibicarius* (IUCN status: Vulnerable), is a semi-aquatic montane ranid frog formerly common throughout the mountain ranges of Tilarán, Cordillera Central, and Talamanca in Costa Rica and western Panama (Savage, 2002). This species forages in dense montane forests and breeds in ponds and shallow bodies of water located in closed and open areas (Savage, 2002). The IUCN status of the species was changed to “Critically Endangered” and “Possibly Extinct” after its population declines and disappearance across the distribution range in the late 1990s (IUCN SSC Amphibian Specialist Group and NatureServe, 2013). The causes of its apparent disappearance are still not clear; however, the declines coincided with the arrival of *Bd*, suggesting *Bd* as one of the main drivers of the declines. Six years after disappearing, the species was re-encountered at a few sites in Costa Rica and reproduced in high numbers despite its co-existence with *Bd* (Whitfield et al., 2017). Since 2013, its IUCN status has been changed to “Vulnerable.”

*Craugastor escoces* [IUCN status: “Extinct”; rediscovered in 2016 (Jiménez and Alvarado, 2017)] is a stream-breeding endemic frog that used to be extremely common throughout the Cordillera Central mountain range in Costa Rica (Savage, 2002). The cause of its presumed extinction is not fully understood; however, chytridiomycosis is suspected to be one of the main causes (Bolaños and Chaves, 2004). This species belongs to the Central American *Craugastor punctariolus* group (Hedges et al., 2008) that exemplifies the severe population declines of montane riparian amphibians throughout Central America. Interestingly, the decline and extirpation of another species from this group, *C. punctariolus*, has been associated with a *Bd*-outbreak (Ryan et al., 2008).

*Isthmohyla rivularis* (IUCN status: “Critically Endangered”) is an arboreal frog associated with fast-flowing highland streams (Savage, 2002). The species was formerly common throughout the mountain ranges of Tilarán, Cordillera Central, and Talamanca in Costa Rica and western Panama, but populations were extirpated. *I. rivularis* was presumed to be extinct until 2007 when it was rediscovered in the Monteverde cloud forest (Wheelwright, 2000). It was recently found in our study site as well (Jiménez et al., 2019). The cause of the decline of this species is not known; however, chytridiomycosis has been suggested as a possible factor involved in population extirpations (Solís et al., 2010).

Lastly, *I. pseudopuma* (IUCN status: “Least Concern”) is an arboreal frog that can be found in both disturbed and undisturbed habitats, and its distribution is similar to that of *L. vibicarius*. It is an explosive breeder, congregating at permanent and temporal ponds in the highlands (Savage, 2002). Individuals infected with *Bd* have been identified (Puschendorf et al., 2009), but the species seems to have evaded population declines (Pounds et al., 1997).

### Sampling and Molecular Detection of *Bd*

In order to describe the variation in the skin microbiome of sympatric species and specifically to investigate the prevalence of bacterial genera with putatively anti-*Bd* members according to *Bd-*infection status, we sampled adults of *L vibicarius*, *I. pseudopuma*, *I. rivularis*, and *C. escoces* (“species data set”). In order to understand the life stage at which (1) the protective skin microbiome is shaped and (2) shifts occur in the abundance of putatively *Bd*-inhibitory bacteria, we sampled tadpoles, juveniles, and adults of *L. vibicarius* (“life-stage data set”). We obtained unequal sample sizes among study sites for each life stage due to adverse conditions during fieldwork, with either few rainy days to stimulate the frogs’ activity or difficulty accessing study sites after heavy rains. Sample sizes for microbiome analyses are given in Supplementary Table S1.

Adult and juvenile frogs were captured and handled with clean gloves, and each frog was kept in an individual clean plastic bag or small container. Tadpoles of similar size and developmental stage (Gosner stage 26–29) were captured with a dip net. Swabbing consisted of moving a sterile rayon-tipped swab (Peel Pouch Dryswab^TM^ Fine Tip) across the animal’s skin following a standard protocol. The swabbing protocol for adults and juveniles consisted of 10 strokes along the ventral side, five strokes along each thigh, and five strokes across each foot. For tadpoles, the protocol consisted of 10 strokes on each side (along body and tail), 10 strokes on the dorsal surface of the body, and 10 strokes on the mouth. Before swabbing, we rinsed every individual with distilled autoclaved water to remove sediment, dirt, and transient bacteria. We swabbed individuals twice using new swabs each time; the first swab was used for identifying skin bacteria and the second to test for *Bd* infection (Familiar López et al., 2017). Swabs for bacterial analysis were stored in 1.5 ml sterile Eppendorf tubes with 300 μl DNA/RNA Shield (Zymo Research), and swabs for *Bd* analysis were placed directly in dry 1.5 ml sterile Eppendorf tubes. All swabs were stored at −20°C until further processing.

To test for the presence of *Bd* in individual samples, we followed previously described qPCR protocols (Boyle et al., 2004; Whitfield et al., 2013). We extracted DNA from swabs using 50 mL of PrepMan Ultra (Applied Biosystems, Foster City, CA, United States) and detected the presence of *Bd* DNA using a TaqMan qPCR assay in a QuantStudio^®^ 3 Real-Time PCR System. Samples were run with one negative control and two positive controls. Positive samples were analyzed in triplicate to verify the result (Whitfield et al., 2013).

### Microbiome Sequencing

We extracted bacterial genomic DNA from swabs using standard protocols for the NucleoSpin Soil Kit (Macherey-Nagel). To yield more DNA, we preheated SE buffer to 37°C and conducted two consecutive elution steps (2 × 45 μl). We followed the 4-primer amplicon tagging scheme of Fluidigm (Access Array System for Illumina Sequencing Systems, Fluidigm Corporation) in which amplicon PCR and barcoding PCR occur simultaneously. For amplification, we used the primers 515F (5′-GTGCCAGCMGCCGCGGTAA-3′) and 806R (5′-GGACTACHVGGGTWTCTAAT-3′), which target a 291-bp fragment of the hypervariable V4 region of the 16S rRNA gene. However, these primers had to be adapted according to the Fluidigm protocol, and so tagged target-specific primers (CS1-TS-515F and CS2-TS-806R) were combined with sample-specific primer pairs that contained a barcoding sequence and the adaptor sequences used by the Illumina sequencing systems. Final 15-μl volumes for the target specific 4-primer amplicon tagging reaction contained 10 ng/μl DNA, 1X FastStart PCR grade nucleotide mix buffer without MgCl_2_ (Roche), 4.5 mM MgCl_2_ (Roche), 200 μM of each PCR grade nucleotide (Roche), 0.05 U/μl FastStart high fidelity enzyme blend (Roche), 1X access array loading reagent (Fluidigm), 400 nM access array barcode primers for Illumina (Fluidigm), 5% DMSO (Roche), 2.4% PCR-certified water, and 50 nM target-specific primers (TS-515F and TS-806R). The barcoded samples were purified by using NucleoMag NGS Beads (Macherey-Nagel) with a 1:1 ratio of amplicons to beads and quantified with PicoGreen on Tecan F200. We then pooled all samples to an equal amount of 12 ng DNA and diluted the pool down to 6 nM. Finally, the library was loaded at 7.5 ppm and sequenced on an Illumina MiSeq instrument by using a 250-bp paired-end strategy with 10% PhiX added to account for low base diversity. Raw sequencing data is available in the Dryad Digital Repository: https://doi.org/10.5061/dryad.n34035p.

### Bioinformatic Analyses

The initial processing of sequencing reads was performed as previously described (Šrut et al., 2019). Sequencing reads were pre-processed with the open-source QIIME2 (version 2019.1) (Bolyen et al., 2018). For dada2 analysis, we trimmed the first bases of each read both to remove primers (–p-trim-left-f 23, –p-trim-left-r 20) and to truncate forward and reverse reads to 200 bp because of decreasing average quality scores of the sequences at the end. Finally, we collapsed reads into representative sequences, known as amplicon sequence variants (ASVs). The ASV approach has been shown to outperform Operational Taxonomic Unit (OTU) clustering; unlike OTU clustering, ASVs are not compared with a reference database and are retained when they meet an arbitrary similarity cutoff (Callahan et al., 2017). We generated a mid-point-rooted bacterial phylogenetic tree for further diversity analyses by using MAFFT (Katoh and Standley, 2013) and constructed a phylogenetic tree using Fast Tree 2 (Price et al., 2010). We assigned taxonomy to the resulting ASVs with the Greengenes database (version 13_8) as a reference^1^. Then, we removed sequences classified as chloroplast, mitochondria, archaea, Eukaryota, and unclassified phylum.

In order to be able to undertake further analyses (i.e., subsequent data filtering and statistical analyses) in the R environment (R Core Team, 2018), the QIIME2 artifacts “feature-table.qza,” “phylogenetic-tree.qza,” and “taxonomy.qza” were converted to .biom, .tsv, and .nwk files, respectively. Finally, we created a new feature-table.biom file (feature-table-taxonomy.biom) by adding the taxonomy information from the “taxonomy.tsv” file to the “feature-table.biom” file.

We used the R package “phyloseq” (McMurdie and Holmes, 2013) to import and merge the “feature-table-taxonomy.biom” file, the bacterial phylogenetic tree, and a text file containing the metadata. We prepared separate datasets according to the analysis, i.e., a “species dataset” and a “life-stage dataset.” In downstream analyses, we excluded ASVs that contained fewer than 50 reads. We also excluded samples with less than 9000 sequences, except for four samples in the “species dataset” [one *C. escoces* sample (4026 reads) and three *L. vibicarius* samples (7081, 8416 and 8999 reads)] because of their relevance for the study. The number of reads per sample ranged from 4026 to 52,154 in the “species dataset” and 9195 and 53,957 in the “life-stage dataset.” For alpha and beta diversity analyses, we normalized read counts across samples by rarefying datasets according to the sample with the lowest read number using the R package “phyloseq.”

### Data Visualization and Statistical Analysis in the R Environment

In order to visualize differences in the relative abundance of skin bacterial taxa, we generated stacked bar charts at the phylum, family, and genus levels. These charts were produced using the R packages “phyloseq” and “ggplot2” (Wickham, 2016).

We calculated two alpha diversity indices, “number of observed ASVs” and “Shannon Index” (Shannon, 1948; Spellerberg and Fedor, 2003), for each skin microbiome sample using the R package “phyloseq.” The Shannon Index is a metric that weights the number of observed ASVs by their relative evenness across the community (Reese and Dunn, 2018).

To test the effect of life stage on the number of observed ASVs and Shannon Index, we performed Generalized Linear Models (GLMs) with Gamma and Gaussian distributions, respectively. We also included site and year as covariates in the models. Both alpha diversity measures were log-transformed prior to analysis to improve normality. We used the odds ratio (OR) and 95% confidence intervals (95% CI) (Nakagawa and Cuthill, 2007), estimated with the R package “emmeans” (Lenth, 2019), to quantify the deviation (i.e., effect size) of the alpha diversity measures between life stages. In pairwise comparisons, an overlap between the 95% CI of the OR estimates and the value “1” indicates that there is no difference.

We assessed the beta diversity of skin bacterial communities across life stages using the unweighted UniFrac distance (based on presence-absence and phylogenetic distance) and the weighted UniFrac distance (based on abundance and phylogenetic distance). These distance matrices were calculated using the R package “phyloseq.” Because these diversity metrics are sensitive to ASVs with very low abundance, we removed low abundance ASVs from the rarefied data by filtering out those with a prevalence lower than 10 (defined as the number of samples in which a taxon appears), and then calculated the distance matrices. We fitted permutational multivariate analyses of variance (PERMANOVAs) using the *adonis* function of the R package “vegan” (Oksanen et al., 2014) to evaluate the variation of the skin bacterial community composition and structure across life stages. We incorporated site and year in PERMANOVAs to account for their variation. We noted the amount of variation explained by the covariates using the *R*^2^ from the output of the *adonis* function. In addition, pairwise PERMANOVAs with Bonferroni corrections were performed to further investigate the effect of life stage on skin bacterial beta diversity. Furthermore, we quantified the extent of the difference in the distances between group centroids (life stages) as a measure of effect size by calculating Cohen’s *d* and 95% CI with the R package “compute.es” (Del Re, 2013). If the 95% CI of Cohen’s *d* estimates overlap with “0,” differences are not considered significant in pairwise comparisons. We performed principal coordinate analysis (PCoA) to visualize the beta diversity distances between life stages.

DESeq2 within the R package “*phyloseq*” was used to identify bacterial taxa (at family and genus levels) that significantly differed in abundance between *L. vibicarius* life stages (adjusted *p*-value < 0.01). We calculated the geometric means of bacterial abundance counts before analysis using the unrarefied dataset. DESeq2 fits negative binomial GLMs for group comparisons and accounts for variation in library size between samples. We used the tax_glom function of the R package “phyloseq” to group ASVs at family and genus levels.

Among the four species as well as among *L. vibicarius* life stages, we explored the presence and abundance of putative anti-*Bd* bacterial ASVs. To do so, we filtered the ASVs table to retain only the reads that matched with members that are known to have anti-*Bd* properties using a database of culturable anti-*Bd* bacteria (Antifungal Isolates Database; Woodhams et al., 2015) as a reference. This database contains 16S rRNA gene sequences isolated from amphibian skin. We retained ASVs with 100% sequence identity match to those in the mentioned database following the methods outlined by Muletz-Wolz et al. (2019). We caution that this does not necessarily mean that these bacterial strains possess anti-*Bd* properties, rather that they are strong candidates. The use of this database to detect putatively anti-*Bd* bacterial strains has been used in previous amphibian microbiome studies (e.g., Kueneman et al., 2015; Abarca et al., 2018; Muletz-Wolz et al., 2019). We visualized the presence and relative abundance patterns with a Venn diagram and heatmaps using the R packages “Vennerable” and “ggplot2,” respectively.

## Results

### Variation in the Skin Microbiome and Prevalence of Putatively Anti-*Bd* ASVs in Adults of Four Sympatric Species

Amplicon sequence variants of the phylum Proteobacteria dominated the skin microbiome of both *Bd–* and *Bd*+ *L. vibicarius* adult frogs (50 and 40%, respectively) and of *Bd*+ *I. rivularis* (82%) and *Bd– C. escoces* (48%) (Figure 1B). In contrast, ASVs of the phylum Bacteroidetes were most abundant on the skin of *Bd– I. pseudopuma* (61%) (Figure 1B).

At the family level, Alcaligenaceae was the most abundant in *Bd– L. vibicarius* (17%), whereas Weeksellaceae (12%) was the most prevalent in *Bd*+ *L. vibicarius* (Figure 1C). The family Weeksellaceae (58%) was also the most dominant taxa in the skin microbiome of *Bd– I. pseudopuma*, followed by Alcaligenaceae (16%). The *Bd*+ *I. rivularis* revealed a markedly different pattern. Most ASVs were assigned to Pseudomonadaceae (29%), tailed by Moraxellaceae (14%) (Figure 1C). The *Bd– C. escoces* showed a microbial pattern with ASVs from 11 assigned families that were evenly distributed (relative abundance <10% each), e.g., *Sphingomonadaceae*, *Sphingobacteriaceae*, and *Pseudomonadaceae* (Figure 1C).

In *L. vibicarius*, with regard to ASVs identified at the genus level, *Bacteroides* and *Pseudomonas* were the most prevalent in *Bd–* individuals (5.4 and 4.2%, respectively), whereas *Cloacibacterium* (10.3%) dominated in *Bd*+ individuals (Supplementary Figure S1). In *Bd– I. pseudopuma*, most ASVs belonged to *Chryseobacterium* (57%) and, in *Bd*+ *I. rivularis*, to *Pseudomonas* (28%). ASVs of the genera *Sphingomonas* and *Pseudomonas* were the most dominant in the *Bd– C. escoces* (8.5 and 5.8%, respectively) (Supplementary Figure S1).

**FIGURE 2:**
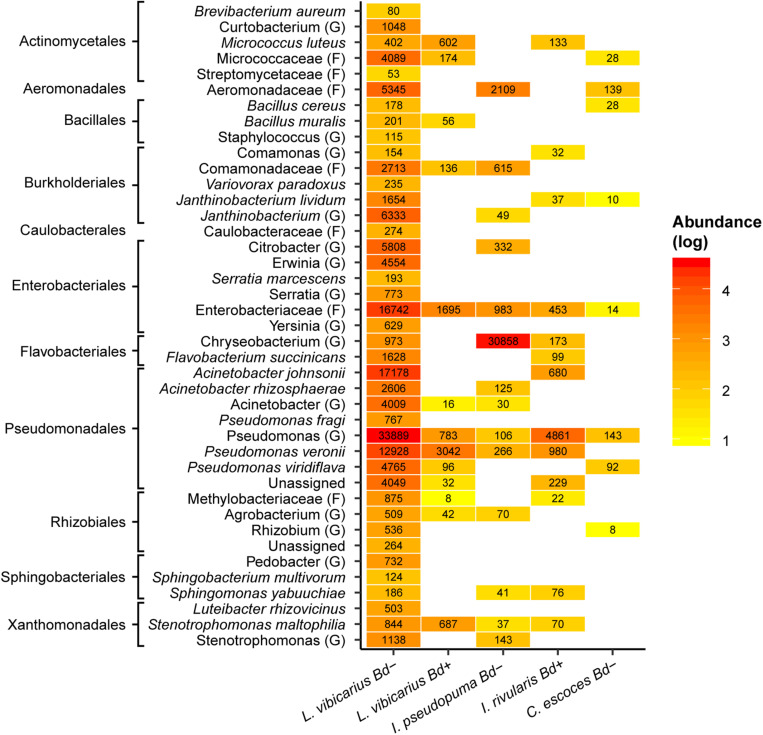
Heatmap of putatively anti-*Bd* ASVs across adults of four sympatric species grouped by *Bd* infection status. Reddish colors indicate increasing abundance, yellowish colors indicate lower abundance, whereas white indicates absence of ASVs. The highest possible taxonomic assignment (maximal at the species level) is shown for these ASVs. The numbers indicate the total number of reads of ASVs assigned to specific taxa. G, genus; F, family.

Among the four sympatric frog species, we detected a total of 94 putative anti-*Bd* ASVs (Supplementary Table S2), from which 77.7 and 31.9% were identified at genus and species levels, respectively. The *Bd– L. vibicarius* showed the highest number of putatively anti-*Bd* ASVs among all samples (Supplementary Table S2). Putatively anti-*Bd* ASVs assigned to the families Enterobacteriaceae and Comamonadaceae, as well as to the genera *Pseudomonas* and *Stenotrophomonas*, were the most frequent and abundant on the skin of both *Bd*+ and *Bd–* frogs (Figure 2). Specifically, in *L. vibicarius*, we observed 91 (96.80%) of these putatively anti-*Bd* ASVs in the skin microbiome of *Bd–* individuals and 19 (20.21%) in *Bd*+ individuals (Supplementary Table S2). Interestingly, putative anti-*Bd* ASVs assigned to *Janthinobacterium lividum*, *Flavobacterium succinicans*, *A. johnsonii*, and *Serratia marcescens* were present only on *Bd– L. vibicarius* (Figure 2). On the skin of both *Bd– I. pseudopuma* and *Bd*+ *I. rivularis*, we observed 17 (18.08%) of these associated potential *Bd–*protective bacterial taxa (Supplementary Table S2). In *Bd– I. pseudopuma*, putatively anti-*Bd* ASVs assigned to *Chryseobacterium* spp. showed the highest abundance, whereas on *Bd*+ *I. rivularis*, potential *Bd*-protective ASVs assigned to *Pseudomonas* spp. and *P. veronii* and *A. johnsonii* were the most abundant (Figure 2). Strikingly, the skin of *Bd– C. escoces* harbored a relatively low number of putatively anti-*Bd* ASVs, with only eight (8.50%) taxa detected (Supplementary Table S2), and those assigned to the family Aeromonadaceae and the genus *Pseudomonas* were detected with the highest abundance (Figure 2).

### Shifts in the Skin Microbiome and Putatively Anti-*Bd* ASVs Across Life Stages of *L. vibicarius*

**FIGURE 3:**
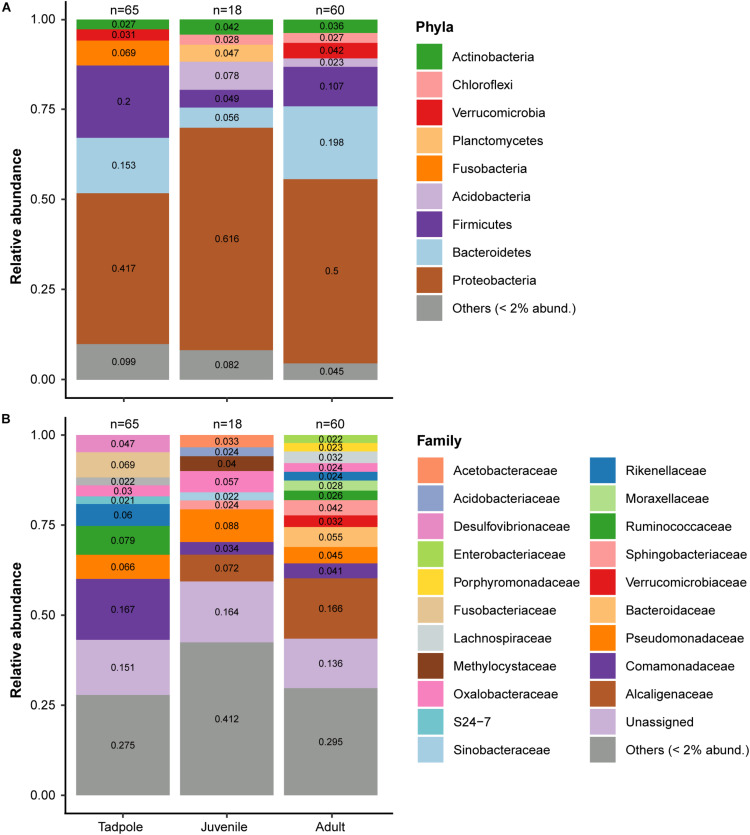
Skin microbiome of *L. vibicarius* across life stages. **(A)** Relative abundance of skin bacterial taxa at the phylum level. Rare ASVs (relative abundances <2%) were clustered together. **(B)** Relative abundance of skin bacterial taxa at the family level. ASVs with relative abundances <2% were clustered together. Only *Bd*– individuals are included.

The skin microbiomes of *L. vibicarius* tadpoles, juveniles, and adults were mainly dominated by ASVs from the phylum Proteobacteria (42, 62, and 50%, respectively). Moreover, ASVs from the phylum Firmicutes and Bacteroidetes were highly abundant (>10%) in both tadpoles and adults but were less frequent in juveniles (Figure 3A). At the family level, Comamonadaceae was the most prominent in the microbiome of tadpole skin (17%), whereas Pseudomonadaceae and Alcaligenaceae were most abundant in juveniles (9 and 7.2%, respectively). In the microbiome of adult skin, the most abundant identified family was Alcaligenaceae (17%) followed by Bacteroidaceae (5.5%) (Figure 3B).

With regard to the alpha diversity of the skin microbiome, we found a strong influence of life stage on the number of observed ASVs (*p* < 0.001) (Supplementary Figure S2A), but not on the Shannon index (*p* = 0.95) (Supplementary Figure S2B). The number of observed ASVs was higher in tadpoles than in juveniles (OR = 1.54, 95% CIs not overlapping 1) and adults (OR = 1.43, 95% CIs not overlapping 1), whereas juveniles and adults showed a similar diversity measure (OR = 0.93, 95% CIs overlapping 1). Both alpha diversity measurements were influenced by year, but not by the study site (Table 1).

**TABLE 1:** Summary of the GLM predicting (a) Number of observed ASVs, (b) Shannon index, according to life stage, study site, and year.

**Variables**	**LR Chi square**	**DF**	***p***
**(a) Number of observed ASVs**			
Life stage	15.67	2	**0.0003**
Site	4.91	3	0.18
Year	13.58	2	**0.001**
**(b) Shannon index**			
Life stage	0.09	2	0.95
Site	1.95	3	0.58
Year	8.48	2	**0.01**

*Significant *p*-values (<0.05) are shown in bold.*

**FIGURE 4:**
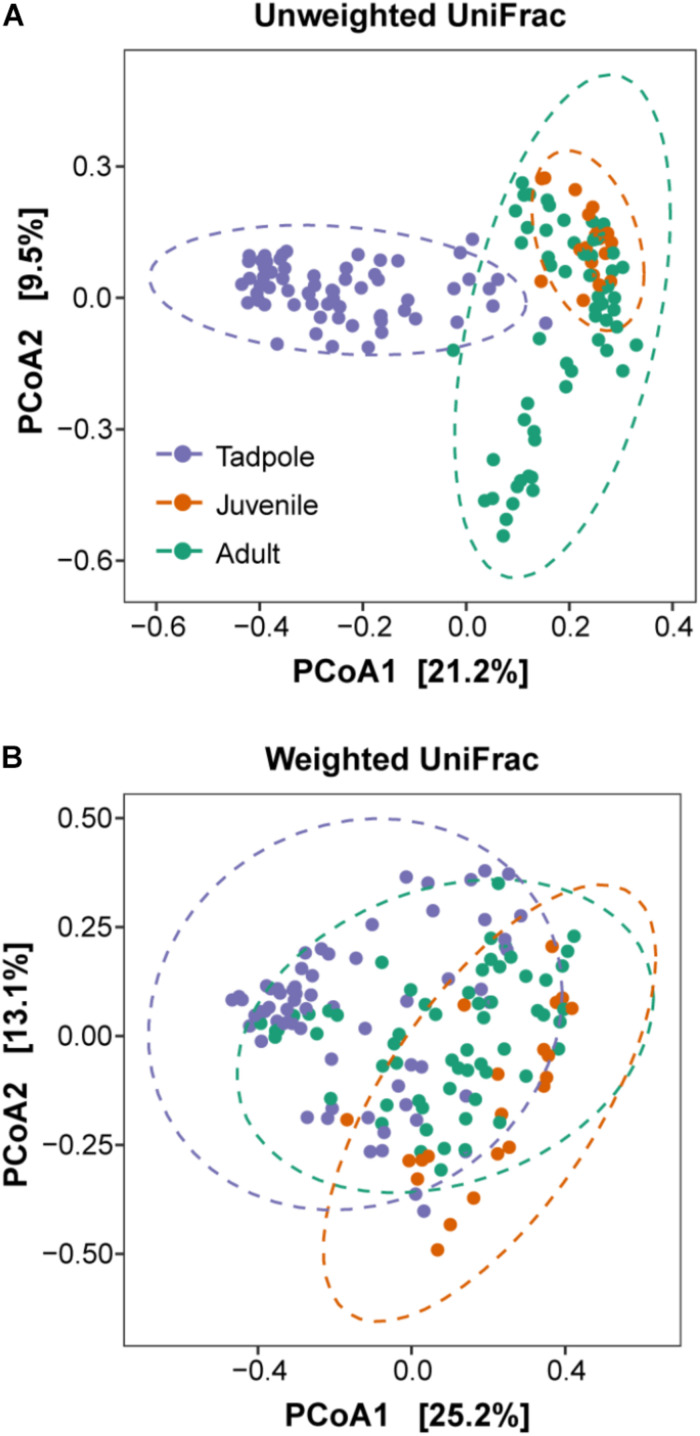
Principal coordinate plots (PCoA) of beta diversity of the skin microbiome composition. **(A)** Unweighted UniFrac matrices and **(B)** Weighted UniFrac matrices of *L. vibicarius* across life stages. Ellipses show 95% confidence intervals (95% CIs) of each life stage. Each point represents the skin bacterial community of an individual sample.

Concerning beta diversity, the PERMANOVA models revealed an effect of life stage on the bacterial community composition (Unweighted UniFrac: *R*^2^ = 0.20, *p* = 0.001) and abundance pattern (Weighted UniFrac: *R*^2^ = 0.17, *p* = 0.001) (Table 2). Moreover, the study site and year explained part of the variation in the skin bacterial communities (Table 2). Pairwise PERMANOVA tests and Cohen’s *d* effect sizes showed that the *R*^2^ and effect sizes between juveniles and adults were much lower, indicating smaller differences between these stages (Table 3). The effects of life stage on the beta diversity indices are visualized by PCoA ordination plots (Figure 4).

**TABLE 2:** Summary of PERMANOVA models of (A) Unweighted and (B) Weighted UniFrac distances according to life stage, study site, and year.

**Variables**	**Sums of squares**	**Mean squares**	**F. model**	***R*^2^**	***p***
**(A) Unweighted UniFrac**					
Life stage	8.44	4.22	19.98	0.21	**0.001**
Site	2.71	0.91	4.28	0.07	**0.001**
Year	1.76	0.88	4.16	0.04	**0.001**
Residuals	28.11	0.21		0.69	
Total	41.04			1	
**(B) Weighted UniFrac**					
Life stage	5.67	2.83	15.27	0.17	**0.001**
Site	2.22	0.74	3.99	0.06	**0.001**
Year	1.60	0.80	4.32	0.04	**0.001**
Residuals	24.68	0.19		0.72	
Total	34.18			1	

*Significant *p*-values (<0.05) are shown in bold.*

**TABLE 3:** PERMANOVA pairwise comparisons of (A) Unweighted and (B) Weighted UniFrac distances between life stages.

**Comparison**	***R*^2^**	***p***	**Adjusted *p***	**Cohen’s *d* (95% CI)**
**(A) Unweighted UniFrac**				
Tadpole–Juvenile	0.18	0.001	0.003	3.47 (2.72–4.23)
Tadpole–Adult	0.18	0.001	0.003	2.19 (1.74–2.65)
Juvenile–Adult	0.06	0.001	0.003	0.92 (0.36–1.48)
**(B) Weighted UniFrac**				
Tadpole–Juvenile	0.16	0.001	0.003	1.60 (1.02–2.19)
Tadpole–Adult	0.12	0.001	0.003	0.89 (0.51–1.28)
Juvenile–Adult	0.07	0.001	0.003	0.84 (0.29–1.41)

**FIGURE 5:**
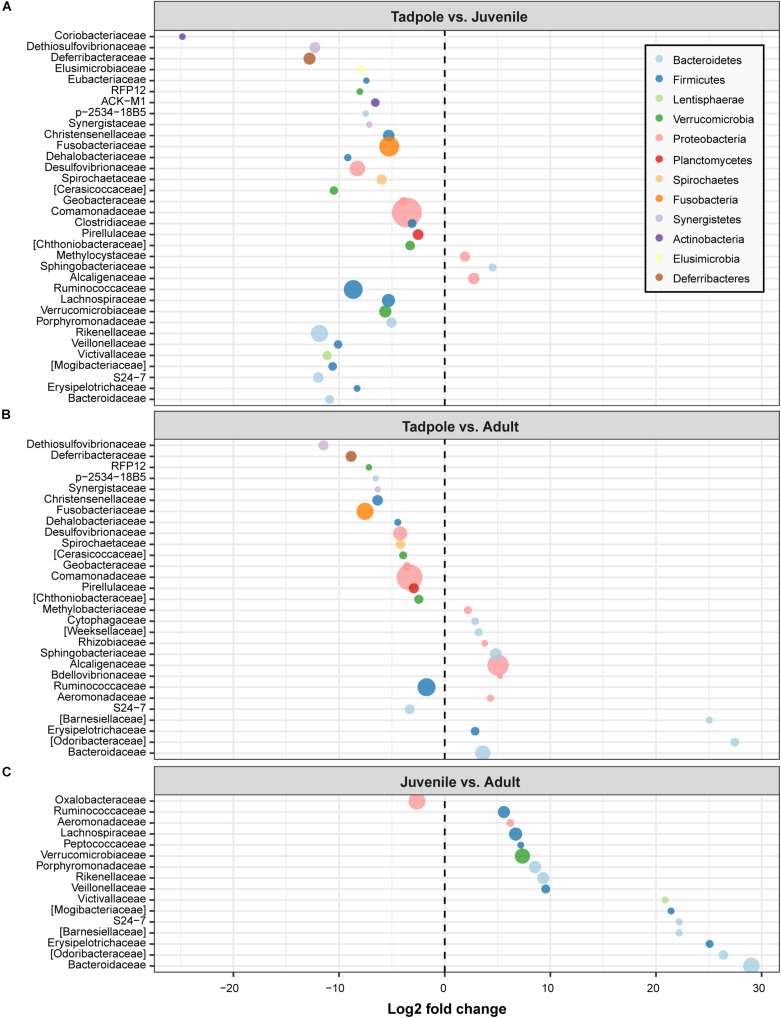
Fold changes of identified bacterial families (circles) that differed between life stages. **(A)** Tadpole vs. juvenile, **(B)** Tadpole vs. adult, and **(C)** Juvenile vs. adult. Bacterial families with a log2 fold change less than 0 were more abundant in the life stage indicated on the left, whereas those with a log2 fold change higher than 0 were more abundant in the indicated life stage on the right. Bacterial families are colored by phylum and sized by mean relative abundance across samples.

DESeq2 analyses revealed important differences in the prevalence and abundance pattern of bacterial families and genera. Families detected in different life stages were in most cases significantly more abundant in tadpoles (Figure 5). In particular, we detected 34 families with significantly different abundances in the skin microbiome of tadpoles and juveniles (Figure 5A). Of these, 31 had a higher abundance in tadpoles, whereas only three exhibited this difference in juveniles. In tadpoles, this aspect especially concerned the families Coriobacteriaceae (log2 fold change: −24.83), Deferribacteraceae (−12.79), Dethiosulfovibrionaceae (−12.29), and S24-7 (−11.97) and, in juveniles, the families Sphingobacteriaceae (4.52), Alcaligenaceae (2.75), and Methylocystaceae (1.91) (Figure 5A). Comparing the skin microbiome of tadpoles and adults, we identified 17 families with higher abundances in tadpoles (e.g., Dethiosulfovibrionaceae, Deferribacteraceae, Fusobacteriaceae, and RFP12), whereas adults showed 12 families with higher abundances, such as [Odoribacteraceae] (log2 fold change: 27.45), [Barnesiellaceae] (25.04), Bdellovibrionaceae (5.24), Alcaligenaceae (5.04), and Sphingobacteriaceae (4.84) (Figure 5B). Lastly, we observed only the bacterial family Oxalobacteraceae (log2 fold change: −2.62) as being more common in juveniles than in adults, whereas 15 families were more abundant in adults, such as Bacteroidaceae (29.02) and Odoribacteraceae (26.38) (Figure 5C).

**FIGURE 6:**
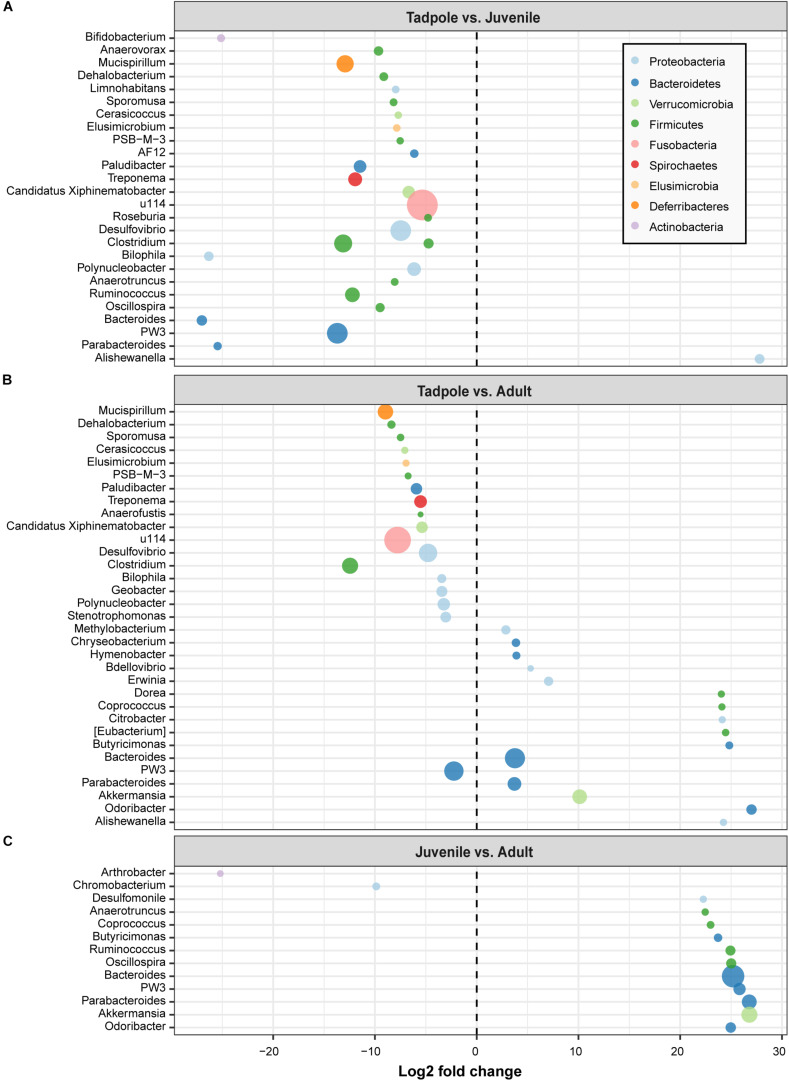
Fold changes of identified bacterial genera (circles) that differed between life stages. **(A)** Tadpole vs. juvenile, **(B)** Tadpole vs. adult, and **(C)** Juvenile vs. adult. Bacterial genera with a log2 fold change less than 0 were more abundant in the life stage indicated on the left, whereas those with a log2 fold change higher than 0 were more abundant in the indicated life stage on the right. Bacterial genera are colored by phylum and sized by mean relative abundance across samples.

When looking at the genus level, we identified 27 genera with significantly different abundance between tadpoles and juveniles (Figure 6A). Of these, 26 genera revealed a higher abundance in tadpoles and only one in juveniles. For instance, the genera *Bacteroides* (log2 fold change: −27.0), *Bilophila* (−26.32), *Parabacteroides* (−25.45), and *Bifidobacterium* (−25.12) showed a very high abundance in tadpoles relative to juveniles (Figure 6A). Furthermore, we identified 18 genera that were more abundant in tadpoles (e.g., *Clostridium* and *Mucispirillum*) than in adults. However, adults showed a higher abundance of 15 genera, such as *Odoribacter* (log2 fold change: 27.0), *Butyricimonas* (24.82), and *Eubacterium* (27.46) (Figure 6B). Moreover, in tadpoles, we found one genus (*Stenotrophomonas*) whose members have been previously associated with possessing anti-*Bd* activity that differed greatly in abundance relative to adults, whereas four genera with anti-*Bd* activity members were detected in adults (*Chryseobacterium*, *Hymenobacter*, *Erwinia*, and *Citrobacter*). The juvenile skin microbiome exhibited a significantly higher abundance of only two genera, namely *Arthrobacter* (log2 fold change: −25.19) and *Chromobacterium* (−9.87), relative to adults, whereas adults harbored higher abundances of 11 genera, such as *Akkermansia* (26.80) and *Parabacteroides* (26.78) (Figure 6C).

**FIGURE 7:**
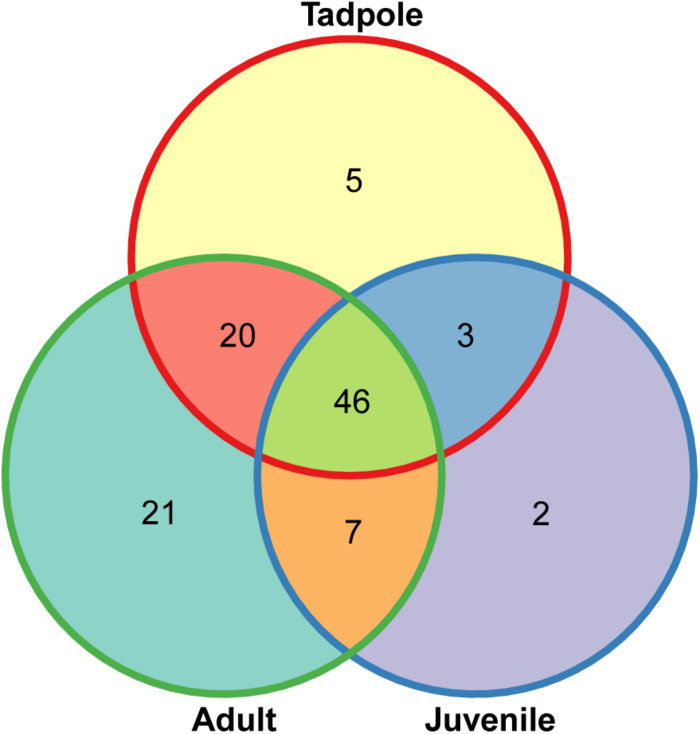
Venn diagram of putative *Bd*-inhibitory ASVs across life stages of *L. vibicarius*.

**FIGURE 8:**
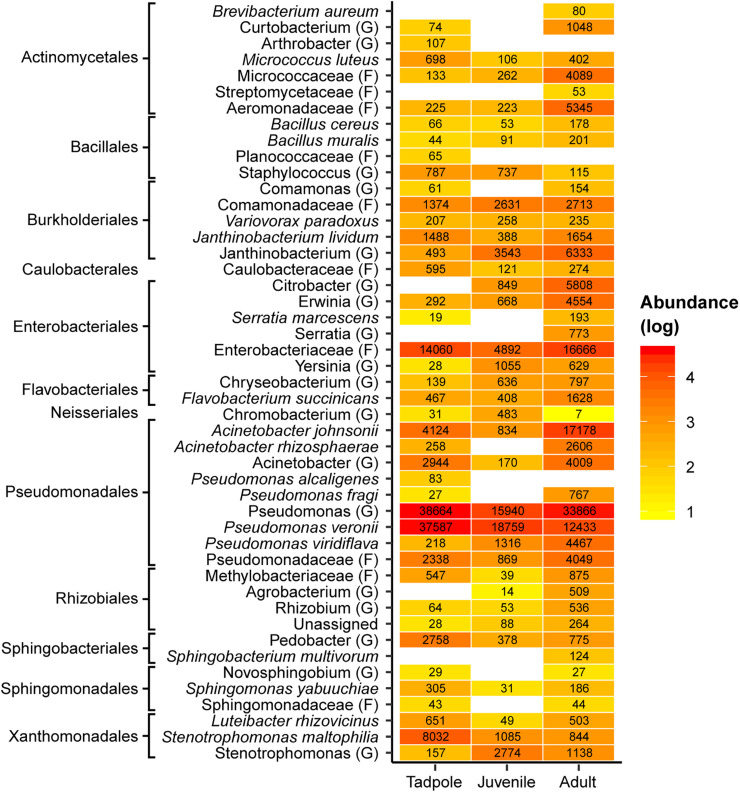
Heatmap of putative *Bd*-inhibitory ASVs across life stages of *L. vibicarius*. Reddish colors indicate increasing abundance, yellowish colors indicate lower abundance, whereas white indicates absence of ASVs. The highest possible taxonomic assignment (maximal at the species level) is shown for these ASVs. The numbers indicate the total number of reads of ASVs assigned to specific taxa. G, genus; F, family.

Putatively anti-*Bd* bacterial ASVs were abundant in both pre- and post-metamorphic stages of *L. vibicarius*. We detected 104 ASVs associated with inhibiting *Bd*-growth across all life stages (Figure 7 and Supplementary Table S3). We found 74 of these in the skin microbiome of tadpoles, whereas 58 and 94 were observed in the skin microbiomes of juveniles and adults, respectively (Figure 7 and Supplementary Table S3). Interestingly, 46 ASVs (48%) with putative anti-*Bd* activity were present in all three life stages, and tadpoles and adults had the highest overlap (66 ASVs, 69%) (Figure 7). The putatively anti-*Bd* ASVs assigned to the family Enterobacteriaceae, the genera *Pseudomonas*, and the species *P. veronii* and A. *johnsonii* were present in all life stages with the highest abundance (Figure 8).

## Discussion

Despite growing evidence that cutaneous bacteria play an important role in amphibian health (reviewed by Jiménez and Sommer, 2017), our current understanding of the microbiome in amphibians from tropical systems remains limited. This is the first study using high-throughput sequencing to investigate the skin microbiome of amphibians that persist in co-existence with *Bd* after their presumed disappearance/extinction from the highlands of Costa Rica. We hypothesized that (1) skin bacterial communities would differ between co-occurring species and would be distinct in *Bd*-infected and *Bd*-uninfected hosts, (2) *Bd*-uninfected individuals would harbor a higher diversity of putatively *Bd*-inhibitory bacterial ASVs, (3) the skin microbiome would differ between life stages of *L. vibicarius*, and (4) the diversity of putatively *Bd*-inhibitory bacteria would be higher after the metamorphosis of *L. vibicarius.* With this study, we have begun to fill the gap in our knowledge of the skin microbiome of species with relict populations and suspected resistance to *Bd*.

As hypothesized, we found distinctly different skin microbiomes between adults of co-habiting species. We observed differences in the composition of ASVs at the phyla, family, and genus levels. For instance, with regard to the microbiome across our *Bd*– amphibians, the most dominant family on the skin of *L. vibicarius* is Alcaligenaceae, whereas on *I. pseudopuma* and *C. escoces*, the most prominent families are Weeksallaceae and Planococcaceae, respectively. Moreover, the genus *Chrysobacterium* is present in higher proportions in *I. pseudopuma* than in *L. vibicarius* and *C. escoces*. Interestingly, we observed a higher proportion of ASVs of the genus *Bacteroides* in *L. vibicarius* compared to the other species. This genus plays an important role in maintaining gut homeostasis (Wexler and Goodman, 2017) and therefore could be relevant for supporting the health of *L. vibicarius*. This species-specific microbiome pattern is congruent with other studies in the tropics, such as in low- and mid-elevations of Costa Rica and Panama (Belden et al., 2015; Rebollar et al., 2016b; Abarca et al., 2018) and in the temperate region (McKenzie et al., 2011; Kueneman et al., 2014; Bletz et al., 2017). The family Alcaligenaceae and genus *Chryseobacterium* were also the most common taxa in the bacterial communities of *Agalychnis callidryas*, *Leptodactylus savagei*, and *Pristimantis ridens* from the lowlands of Costa Rica (Abarca et al., 2018). In this study, we have not evaluated the mechanisms driving variation in the skin microbiome; however, we suspect that a distinct antimicrobial peptide composition among our species, as has been observed in other amphibians, plays a role as a selective force that determines which microbial genotypes can grow on each host’s skin (Woodhams et al., 2007a; Franzenburg et al., 2013). This could be driven by differences in ecological and behavioral traits across species (e.g., forest forager vs. arboreal and stream forager). Such differences lead to exposure to distinct environmental conditions (e.g., pH, water temperature), which may result in the stimulation or inhibition of the growth of certain bacterial strains on the skin. Furthermore, the presence of different environmental microbes across the microhabitats used by the species could drive distinct microbial colonization of the skin (Loudon et al., 2014; Bletz et al., 2017).

Considering the *Bd*-infection status, we observed, as expected, a different bacterial composition at the phylum and family levels between *Bd* infected/uninfected adults of the same species, for example in *Bd*– versus *Bd*+ of *L. vibicarius*. We also observed a higher diversity of ASVs with putatively anti-*Bd* activity on the skin of *L. vibicarius Bd*– individuals than on the skin of *Bd*+ individuals. More than half of these ASVs are absent in *Bd*+ individuals, and most of the shared bacterial taxa, such as *P. veronii*, are more prevalent on the skin of *Bd*– individuals than on *Bd*+ individuals. The bacterial composition and higher diversity of putatively beneficial bacteria detected on the skin of *Bd*– *L. vibicarius* suggests that the bacterial community can promote the health of *Bd*– individuals. This observation might be linked to a reduction of the bacterial richness on the skin of highly *Bd*-infected amphibians, as observed in a previous study (Ellison et al., 2018). Further research is needed to confirm this relationship. We should also consider that the observed differences in the skin microbiome might be a direct effect of *Bd*, as found in previous studies on the skin microbiome of *Rana sierrae* and *Lithobates catesbeianus* (Jani and Briggs, 2014; Walke et al., 2015; Jani et al., 2017).

Furthermore, we found bacterial ASVs with putatively anti-*Bd* activity in all four species, regardless of their *Bd*-infection status. Putatively anti-*Bd* ASVs assigned to the genera *Pseudomonas* and *Chryseobacterium* are among the most abundant *Bd*-protective bacteria on *Bd*– *I. pseudopuma* and *Bd*– *C. escoces*, respectively. These two genera have been reported with a high proportion of isolates inhibiting *Bd*-growth (Becker et al., 2015). Notably, we observed less than half of these beneficial bacteria (e.g., putatively anti-*Bd* ASVs assigned to *Pseudomonas* spp. and *A. johnsonii*) from the overall *Bd*-protective bacteria detected across our samples on the skin of *Bd*+ *I. rivularis*. These ASVs might limit the impacts of *Bd*-infection, given that no signs of disease have (so far) been observed in this species. We also detected potentially beneficial ASVs assigned to the genus *Janthinobacterium* on *Bd*– *L. vibicarius*, *Bd*– *I. pseudopuma*, *Bd*– *C. escoces*, and *Bd*+ *I. rivularis*. One species in this genus, *J. lividum*, detected in our study species, is known to inhibit *Bd* efficiently on the salamander *Plethodon cinereus* (Brucker et al., 2008), although this inhibition appears not to be effective in the frog *Atelopus zeteki* (Becker et al., 2011). Given the devastating impact of *Bd* on our endangered focal species [and also in other populations in Costa Rica (Wheelwright, 2000; Whitfield et al., 2017)], these findings support the hypothesis that *Bd*-persistent species possess a skin microbiome with potential for protecting against *Bd*. Because *Bd*– *C. escoces* has a low percentage of ASVs with putatively anti-*Bd* activity, one can speculate that this species has a skin microbiome with a lower ability to fight *Bd*. Notwithstanding, this microbiome conformation might be sufficient to protect the species in conjunction with other mechanisms, such as antimicrobial peptides (Woodhams et al., 2007a). Moreover, the protective role of skin bacteria through the production of antifungal metabolites can be a consequence of cooperation and competition among bacteria (Foster et al., 2017). Nevertheless, because *C. escoces* is a critically endangered species and might remain on the brink of extinction, further research is urgently required to improve our understanding of the species’ skin microbiome and its association to *Bd*-resistance. The use of culture-independent techniques, like the 16S rRNA amplicon sequencing used in this study, to investigate bacterial communities has led to an important improvement in our understanding of amphibian microbial ecology and its interaction with *Bd* (Rebollar et al., 2016a; Jiménez and Sommer, 2017). However, in the light of the high variability of *Bd*-inhibitory functions among genera, within bacterial isolates from a single genus, and even at the strain level (Becker et al., 2015), further inhibition tests with culture-dependent techniques are necessary (1) to confirm anti-*Bd* activity of these beneficial bacteria among distinct hosts and (2) to reach more robust conclusions regarding the protective role of these bacteria against *Bd*-infection. For instance, in three Neotropical frogs (*A. callidryas*, *Dendropsophus ebraccatus*, and *Craugastor fitzingeri*) the majority of the culture-dependent skin bacteria represented in the culture-independent community were *Bd*-inhibitory (Rebollar et al., 2019). Also, further studies are required to evaluate the degree of protection given by microbiomes in our focal species.

In order to understand the life stage at which the protective skin microbiome is shaped and when shifts occur in abundance of putatively *Bd*-inhibitory bacteria, we sampled tadpoles, juveniles, and adults of *L. vibicarius*. We found that life stage is a strong predictor of both alpha diversity (number of observed ASVs, but not Shannon diversity) and beta diversity (Unweighted and Weighted UniFrac distances) of the skin microbiome of *L. vibicarius.* We also detected differences in the abundance of certain skin bacterial families and genera across life stages via DESeq2 analysis. Our findings clearly support the hypothesis that amphibian life stage influences the skin microbiome (Kueneman et al., 2015). Previous research in amphibians, e.g., *Anaxyrus boreas* and *Eleutherodactylus coqui*, has revealed life stage as one of the most important factors structuring microbial communities on amphibian skin (Kueneman et al., 2014, 2015; Longo et al., 2015). Within this study, we have mainly observed strong differences between pre- and post-metamorphic stages, indicating a dramatic restructuring of the skin bacterial community after metamorphosis. We found a large number of observed ASVs in tadpoles relative to juveniles and adults, whereas these last two stages did not differ from one another. Also, we observed different community compositions and abundance patterns between the three life stages. Furthermore, effect sizes showed that differences between tadpoles and the two post-metamorphic stages, namely juveniles and adults, are much stronger than between the two later stages. Indeed, the beta diversity metrics suggest that the core microbiomes of juveniles and adults are more similar to each other than to the microbiome of tadpoles. Despite an incomplete understanding of the mechanisms shaping the skin microbiome across development, the variation in the skin microbiome across life stages is probably attributable to a distinct type of skin; keratinized skin tissue after metamorphosis is likely more suitable for distinct bacterial taxa (Kueneman et al., 2015). Moreover, across amphibian development, the skin’s defensive glands might shift the production of specific antimicrobial peptides that influence the growth of distinct bacterial taxa (Rollins-Smith, 2009). Further research should explore the microbiome diversity of *L. vibicarius* on metamorphs, because metamorphosis represents a switch-point with many drastic physiological and immunological re-arrangements, a period during which amphibians might be highly susceptible to pathogens.

In addition to the strong effect of life stage on beta diversity, we have also observed that the study site and year partly explain the variation in distance metrics. These results are congruent with those observed in earlier studies (McKenzie et al., 2011; Longo et al., 2015; Familiar López et al., 2017). Further studies should better explore the role of environment (i.e., study site) and year in order to unravel the relative importance of these covariates in our study species. In particular, some of our sampling sites are under the constant influence of human activities that could either stress hosts and/or modify environmental bacterial taxa that might colonize amphibian skin (Krynak et al., 2016; Huang et al., 2018). These effects could ultimately impact the skin microbiome homeostasis of the amphibians.

Contrary to our expectations, we did not observe a significant increase in the diversity of putatively anti-*Bd* bacteria after metamorphosis in *L. vibicarius*, as both pre- and post-metamorphic stages of *L. vibicarius* hosted a high number of putatively anti-*Bd* members. The presence of these beneficial bacteria might, at least in part, explain the persistence of this endangered species, now co-existing with *Bd* well after its presumed extinction. These anti-*Bd* members can reach high population densities on amphibian skin (Harris et al., 2009). In addition, we observed that a high percentage of these bacterial members are shared among life stages. These findings suggest that tadpoles, which usually have a low susceptibility to *Bd* and can harbor non-lethal infections, maintain a diverse assemblage of putatively anti-*Bd* bacterial strains. Tadpoles may obtain these putatively anti-*Bd* members through horizontal transmission, and subsequently act as reservoir hosts of many of these bacteria for post-metamorphic life stages with keratinized skin tissues vulnerable to *Bd* infection (Walke et al., 2011; Rebollar et al., 2016c). This is particularly relevant because post-metamorphic frogs in the current study were colonized by many putative anti-*Bd* bacterial ASVs (e.g., *Pseudomonas* spp. and *Janthinobacterium* spp.) Taxonomically diverse assemblages of anti-*Bd* bacteria are more effective at suppressing *Bd* growth than individual bacterial isolates (Piovia-Scott et al., 2017; Bell et al., 2018). We observed a lower number of anti-*Bd* ASVs on the skin of juveniles than on adults, and some bacterial strains were only detected in adults (e.g., *Acinetobacter rhizosphaerae* and *Pseudomonas fragi*). Adaptive immune functions such as the production of skin antimicrobial peptides and alkaloids continue to develop after metamorphosis in association with skin shedding (Rollins-Smith, 2009; Kueneman et al., 2015). These factors may influence the *Bd*-protective bacterial assemblage on juvenile skin and thus explain why juveniles had less putatively anti-*Bd* ASVs than adults (and may be more susceptible to *Bd*). Further work is needed to comprehend bacteria/*Bd* interaction patterns across developmental stages.

The current study extends our knowledge about the skin microbiome in Neotropical montane amphibians, particularly with regard to those species that are *Bd*-susceptible and have relict populations persisting and recovering after dramatic *Bd*-associated declines. Despite a limited sample size, our findings point to the presence of a potential *Bd*-protective microbiome in these endangered species. Our results may also contribute to the development of innovative conservation strategies, such as anti-*Bd* probiotic treatments, to protect susceptible Neotropical amphibian species from the devastating effects of *Bd.* Further research should focus on the genes that characterize microbiome function to advance our understanding of amphibian adaptation and persistence in the environment as well as maintenance of a good health status. Given the observed patterns in skin microbiome diversity across developmental stages and *Bd*-infection status in *L. vibicarius*, we suggest that the microbiome of this frog is an outstanding model for future research.

## Data Availability

Raw sequencing data is available in the Dryad Digital Repository: https://doi.org/10.5061/dryad.n34035p.

## Ethics Statement

Research permits and sampling and animal care protocols were approved by Costa Rica MINAE-SINAC (SINAC-ACAHN-PI-R-004-2015) and CONAGEBio (R-055-2015-OT-CONAGEBio).

## Author Contributions

RJ, SS, and GA contributed to the research design. RJ, JE, and GA performed the sample collection. RJ carried out the laboratory work on microbiome samples, performed the bioinformatic analysis and statistical analysis, and wrote the manuscript with input from SS, GA, and JE. GA and JE carried out the laboratory work on *Bd* samples.

## Conflict of Interest Statement

The authors declare that the research was conducted in the absence of any commercial or financial relationships that could be construed as a potential conflict of interest.

## Footnotes

1 http://greengenes.lbl.gov

## References

[B1] Abarca J. G. Vargas G. Zuniga I. Whitfield S. M. Woodhams D. C. Kerby J. (2018). Assessment of bacterial communities associated with the skin of costa rican amphibians at la selva biological station. *Front. Microbiol.* 9:2001. 10.3389/fmicb.2018.02001 30233511 PMC6129598

[B2] Alibardi L. (2001). Keratinization in the epidermis of amphibians and the lungfish: comparison with amniote keratinization. *Tissue Cell* 33 439–449. 10.1054/tice.2001.0198 11949780

[B3] Becker M. H. Harris R. N. Minbiole K. P. C. Schwantes C. R. Rollins-Smith L. A. Reinert L. K. (2011). Towards a better understanding of the use of probiotics for preventing chytridiomycosis in Panamanian golden frogs. *Ecohealth* 8 501–506. 10.1007/s10393-012-0743-0 22328095

[B4] Becker M. H. Walke J. B. Cikanek S. Savage A. E. Mattheus N. Santiago C. N. (2015). Composition of symbiotic bacteria predicts survival in Panamanian golden frogs infected with a lethal fungus. *Proc. Biol. Sci. U.S.A.* 282:20142881. 10.1098/rspb.2014.2881 25788591 PMC4389611

[B5] Belden L. K. Hughey M. C. Rebollar E. A. Umile T. P. Loftus S. C. Burzynski E. A. (2015). Panamanian frog species host unique skin bacterial communities. *Front. Microbiol.* 6:1171. 10.3389/fmicb.2015.01171 26579083 PMC4621460

[B6] Bell S. C. Garland S. Alford R. A. (2018). Increased numbers of culturable inhibitory bacterial taxa may mitigate the effects of *Batrachochytrium dendrobatidis* in australian wet tropics frogs. *Front. Microbiol.* 9:1604. 10.3389/fmicb.2018.01604 30072970 PMC6058028

[B7] Berger L. Speare R. Daszak P. Green D. E. Cunningham A. A. Goggin C. L. (1998). Chytridiomycosis causes amphibian mortality associated with population declines in the rain forests of Australia and Central America. *PNAS* 95 9031–9036. 10.1073/pnas.95.15.9031 9671799 PMC21197

[B8] Blaustein A. R. Romansic J. M. Scheessele E. A. Han B. A. Pessier A. P. Longcore J. E. (2005). Interspecific variation in susceptibility of frog tadpoles to the pathogenic fungus *Batrachochytrium dendrobatidis*. *Conserv. Biol.* 19 1460–1468. 10.1111/j.1523-1739.2005.00195.x

[B9] Bletz M. C. Archer H. Harris R. N. McKenzie V. J. Rabemananjara F. C. E. Rakotoarison A. (2017). Host ecology rather than host phylogeny drives amphibian skin microbial community structure in the biodiversity hotspot of Madagascar. *Front. Microbiol.* 8:530. 10.3389/fmicb.2017.01530 28861051 PMC5563069

[B10] Bolaños F. (2009). Situación de los anfibios de Costa Rica. *Biocenosis* 22 95–108.

[B11] Bolaños F. Chaves G. (2004). Craugastor escoces. *IUCN Red List Threat. Species* 2004:e.T56588A11488977. 10.2305/IUCN.UK.2004.RLTS.T56588A11488977.en

[B12] Bolyen E. Rideout J. R. Dillon M. R. Bokulich N. A. Abnet C. Al-Ghalith G. A. (2018). QIIME 2: reproducible, interactive, scalable, and extensible microbiome data science. *PeerJ. Inc.* 6:e27295v2. 10.1038/s41587-019-0209-9 PMC7015180 31341288

[B13] Boyle D. G. Boyle D. B. Olsen V. Morgan J. A. T. Hyatt A. D. (2004). Rapid quantitative detection of chytridiomycosis (*Batrachochytrium dendrobatidis*) in amphibian samples using real-time Taqman PCR assay. *Dis. Aquat. Org.* 60 141–148. 10.3354/dao060141 15460858

[B14] Brucker R. M. Harris R. N. Schwantes C. R. Gallaher T. N. Flaherty D. C. Lam B. A. (2008). Amphibian chemical defense: antifungal metabolites of the microsymbiont *Janthinobacterium lividum* on the salamander *Plethodon cinereus*. *J. Chem. Ecol.* 34 1422–1429. 10.1007/s10886-008-9555-9557 18949519

[B15] Callahan B. J. McMurdie P. J. Holmes S. P. (2017). Exact sequence variants should replace operational taxonomic units in marker-gene data analysis. *ISME J.* 11 2639–2643. 10.1038/ismej.2017.119 28731476 PMC5702726

[B16] Castro-Cruz A. García-Fernández F. (2012). Report on *Lithobates vibicarius* (Cope 1894) (*Anura*: *Ranidae*) in Parque Nacional de Agua Juan Castro Blanco. *Alajuela Costa Rica. Froglog.* 100 69–70.

[B17] Chaves G. Zumbado-Ulate H. García-Rodríguez A. Gómez E. Vredenburg V. T. Ryan M. J. (2014). Rediscovery of the critically endangered streamside frog, *Craugastor taurus* (Craugastoridae), in Costa Rica. *Trop. Conserv. Sci.* 7 628–638. 10.1177/194008291400700404

[B18] Collins J. P. Storfer A. (2003). Global amphibian declines: sorting the hypotheses. *Divers. Distrib.* 9 89–98. 10.1046/j.1472-4642.2003.00012.x

[B19] Crump M. L. Hensley F. R. Clark K. L. (1992). Apparent decline of the golden toad: underground or extinct? *Copeia* 1992 413–420. 10.2307/1446201

[B78] De León M. E. Zumbado-Ulate H. García-Rodríguez A. Alvarado G. Sulaeman H. Bolaños F. (2018). Prevalence of the fungal pathogen *Batrachochytrium dendrobatidis* in amphibians of costa rica predated first-known epizootic. *bioRxiv* 482968. 10.1101/482968 PMC6903748 31821326

[B20] Del Re A. C. (2013). *Compute.es**: Compute Effect Sizes. R package version 0.2-2.* Available online at: http://cran.r-project.org/web/packages/compute.es (accessed January 10, 2019).

[B21] Ellison S. Knapp R. A. Sparagon W. Swei A. Vredenburg V. T. (2018). Reduced skin bacterial diversity correlates with increased pathogen infection intensity in an endangered amphibian host. *Mol. Ecol.* 28 127–140. 10.1111/mec.14964 30506592

[B22] Familiar López M. Rebollar E. A. Harris R. N. Vredenburg V. T. Hero J.-M. (2017). Temporal variation of the skin bacterial community and *Batrachochytrium dendrobatidis* infection in the terrestrial cryptic frog *Philoria loveridgei*. *Front. Microbiol.* 8:2535. 10.3389/fmicb.2017.02535 29312226 PMC5744006

[B23] Foster K. R. Schluter J. Coyte K. Z. Rakoff-Nahoum S. (2017). The evolution of the host microbiome as an ecosystem on a leash. *Nature* 548 43–51. 10.1038/nature23292 28770836 PMC5749636

[B24] Franzenburg S. Walter J. Künzel S. Wang J. Baines J. F. Bosch T. C. G. (2013). Distinct antimicrobial peptide expression determines host species-specific bacterial associations. *Proc. Natl. Acad. Sci. U.S.A.* 110 E3730–E3738. 10.1073/pnas.1304960110 24003149 PMC3785777

[B25] González-Maya J. F. Belant J. L. Wyatt S. A. Schipper J. Cardenal J. Corrales D. (2013). Renewing hope: the rediscovery of *Atelopus varius* in Costa Rica. *Amphib. Reptile.* 34 573–578. 10.1163/15685381-15682910

[B26] Grogan L. F. Cashins S. D. Skerratt L. F. Berger L. McFadden M. S. Harlow P. (2018). Evolution of resistance to chytridiomycosis is associated with a robust early immune response. *Mol. Ecol.* 27 919–934. 10.1111/mec.14493 29337419

[B27] Harris R. N. Brucker R. M. Walke J. B. Becker M. H. Schwantes C. R. Flaherty D. C. (2009). Skin microbes on frogs prevent morbidity and mortality caused by a lethal skin fungus. *ISME J.* 3 818–824. 10.1038/ismej.2009.27 19322245

[B28] Harris R. N. James T. Y. Lauer A. Simon M. A. Patel A. (2006). Amphibian pathogen *Batrachochytrium dendrobatidis* is inhibited by the cutaneous bacteria of amphibian species. *Eco. Health* 3:53. 10.1007/s10393-005-0009-1

[B29] Hedges S. B. Duellman W. E. Heinicke M. P. (2008). New World direct-developing frogs (*Anura*: *Terrarana*): molecular phylogeny, classification, biogeography, and conservation. *Zootaxa* 1737 1–82.

[B30] Huang B.-H. Chang C.-W. Huang C.-W. Gao J. Liao P.-C. (2018). Composition and functional specialists of the gut microbiota of frogs reflect habitat differences and agricultural activity. *Front. Microbiol.* 8:2670. 10.3389/fmicb.2017.02670 29375532 PMC5768659

[B31] IUCN SSC Amphibian Specialist Group, and NatureServe (2013). Lithobates vibicarius. *IUCN Red List Threat. Species* 2013:e.T58746A3072875. 10.2305/IUCN.UK.2013-1.RLTS.T58746A3072875.en

[B32] Jani A. J. Briggs C. J. (2014). The pathogen *Batrachochytrium dendrobatidis* disturbs the frog skin microbiome during a natural epidemic and experimental infection. *PNAS* 111 E5049–E5058. 10.1073/pnas.1412752111 25385615 PMC4250152

[B33] Jani A. J. Knapp R. A. Briggs C. J. (2017). Epidemic and endemic pathogen dynamics correspond to distinct host population microbiomes at a landscape scale. *Proc. Biol. Sci. U.S.A.* 284:20170944. 10.1098/rspb.2017.0944 28637861 PMC5489737

[B34] Jiménez R. Alvarado G. (2017). *Craugastor escoces* (Anura: Craugastoridae) reappears after 30 years: rediscovery of an “extinct” Neotropical frog. *Amphib. Reptile.* 38 257–259. 10.1163/15685381-15683102

[B35] Jiménez R. Ballestero E. Astorga J. D. Rodríguez E. Alvarado G. (2019). Isthmohyla rivularis. *Herpetol. Rev.* 50:322.

[B36] Jiménez R. R. Sommer S. (2017). The amphibian microbiome: natural range of variation, pathogenic dysbiosis, and role in conservation. *Biodivers. Conserv.* 26 763–786. 10.1007/s10531-016-1272-x

[B37] Katoh K. Standley D. M. (2013). MAFFT multiple sequence alignment software version 7: improvements in performance and usability. *Mol. Biol. Evol.* 30 772–780. 10.1093/molbev/mst010 23329690 PMC3603318

[B38] King K. C. Brockhurst M. A. Vasieva O. Paterson S. Betts A. Ford S. A. (2016). Rapid evolution of microbe-mediated protection against pathogens in a worm host. *ISME J.* 10 1915–1924. 10.1038/ismej.2015.259 26978164 PMC5029159

[B39] Krynak K. L. Burke D. J. Benard M. F. (2016). Landscape and water characteristics correlate with immune defense traits across Blanchard’s cricket frog (*Acris blanchardi*) populations. *Biol. Conserv.* 193 153–167. 10.1016/j.biocon.2015.11.019

[B40] Kueneman J. G. Parfrey L. W. Woodhams D. C. Archer H. M. Knight R. McKenzie V. J. (2014). The amphibian skin-associated microbiome across species, space and life history stages. *Mol. Ecol.* 23 1238–1250. 10.1111/mec.12510 24171949

[B41] Kueneman J. G. Woodhams D. C. Van Treuren W. Archer H. M. Knight R. McKenzie V. J. (2015). Inhibitory bacteria reduce fungi on early life stages of endangered colorado boreal toads (*Anaxyrus boreas*). *ISME J.* 10 934–944. 10.1038/ismej.2015.168 26565725 PMC4796932

[B42] Langhammer P. F. Burrowes P. A. Lips K. R. Bryant A. B. Collins J. P. (2014). Susceptibility to the amphibian chytrid fungus varies with ontogeny in the direct-developing frog. *Eleutherodactylus coqui*. *J. Wildl. Dis.* 50 438–446. 10.7589/2013-10-268 24807186

[B43] Lemieux-Labonté V. Simard A. Willis C. K. R. Lapointe F.-J. (2017). Enrichment of beneficial bacteria in the skin microbiota of bats persisting with white-nose syndrome. *Microbiome* 5:115. 10.1186/s40168-017-0334-y 28870257 PMC5584028

[B44] Lenth R. (2019). *Emmeans: Estimated Marginal Means, aka Least-Squares Means. R package version 1.3.2.* Available online at: https://CRAN.R-project.org/package=emmeans (accessed January 15, 2019).

[B45] Longo A. V. Savage A. E. Hewson I. Zamudio K. R. (2015). Seasonal and ontogenetic variation of skin microbial communities and relationships to natural disease dynamics in declining amphibians. *Royal Soc. Open Sci.* 2 140377–140377. 10.1098/rsos.140377 26587253 PMC4632566

[B46] Loudon A. H. Woodhams D. C. Parfrey L. W. Archer H. Knight R. McKenzie V. (2014). Microbial community dynamics and effect of environmental microbial reservoirs on red-backed salamanders (*Plethodon cinereus*). *ISME J.* 8 830–840. 10.1038/ismej.2013.200 24335825 PMC3960541

[B47] McFall-Ngai M. Hadfield M. G. Bosch T. C. G. Carey H. V. Domazet-Lošo T. Douglas A. E. (2013). Animals in a bacterial world, a new imperative for the life sciences. *PNAS* 110 3229–3236. 10.1073/pnas.1218525110 23391737 PMC3587249

[B48] McKenzie V. J. Bowers R. M. Fierer N. Knight R. Lauber C. L. (2011). Co-habiting amphibian species harbor unique skin bacterial communities in wild populations. *ISME J.* 6 588–596. 10.1038/ismej.2011.129 21955991 PMC3280140

[B49] McMurdie P. J. Holmes S. (2013). phyloseq: an R package for reproducible interactive analysis and graphics of microbiome census data. *PLoS One* 8:e61217. 10.1371/journal.pone.0061217 23630581 PMC3632530

[B50] Muletz-Wolz C. R. Almario J. G. Barnett S. E. DiRenzo G. V. Martel A. Pasmans F. (2017a). Inhibition of fungal pathogens across genotypes and temperatures by amphibian skin bacteria. *Front. Microbiol.* 8:1551. 10.3389/fmicb.2017.01551 28871241 PMC5566582

[B51] Muletz-Wolz C. R. DiRenzo G. V. Yarwood S. A. Campbell Grant E. H. Fleischer R. C. Lips K. R. (2017b). Antifungal bacteria on woodland salamander skin exhibit high taxonomic diversity and geographic variability. *Appl. Environ. Microbiol* 83:e00186.17. 10.1128/AEM.00186-17 28213545 PMC5394319

[B52] Muletz-Wolz C. R. Fleischer R. C. Lips K. R. (2019). Fungal disease and temperature alter skin microbiome structure in an experimental salamander system. *Mol. Ecol.* 28 2917–2931. 10.1111/mec.15122 31066947

[B53] Nakagawa S. Cuthill I. C. (2007). Effect size, confidence interval and statistical significance: a practical guide for biologists. *Biol. Rev.* 82 591–605. 10.1111/j.1469-185x.2007.00027.x 17944619

[B54] Oksanen J. Guillaume Blanchet F. Kindt R. Legendre P. Minchin P. R. O’Hara R. B. (2014). *vegan: Community Ecology Package. R package Version 2.2-0.* Available online at: http://CRAN.R-project.org/package=vegan (accessed January 10, 2019).

[B55] Park S. T. Collingwood A. M. St-Hilaire S. Sheridan P. P. (2014). Inhibition of *Batrachochytrium dendrobatidis* caused by bacteria isolated from the skin of boreal toads, *Anaxyrus* (*Bufo*) *boreas boreas*, from Grand Teton National Park, Wyoming, USA. *Microbiol. Insights.* 7 1–8. 10.4137/MBI.S13639 24826077 PMC4019225

[B56] Piovia-Scott J. Rejmanek D. Woodhams D. C. Worth S. J. Kenny H. McKenzie V. (2017). Greater species richness of bacterial skin symbionts better suppresses the amphibian fungal pathogen *Batrachochytrium dendrobatidis*. *Microb. Ecol.* 74 217–226. 10.1007/s00248-016-0916-914 28064360

[B57] Pita L. Rix L. Slaby B. M. Franke A. Hentschel U. (2018). The sponge holobiont in a changing ocean: from microbes to ecosystems. *Microbiome* 6:46. 10.1186/s40168-018-0428-421 29523192 PMC5845141

[B58] Pounds J. A. Fogden M. P. L. Savage J. M. Gorman G. C. (1997). Tests of null models for amphibian declines on a tropical mountain. *Conserv. Biol.* 11 1307–1322. 10.1046/j.1523-1739.1997.95485.x

[B59] Prest T. L. Kimball A. K. Kueneman J. G. McKenzie V. J. (2018). Host-associated bacterial community succession during amphibian development. *Mol. Ecol.* 27 1992–2006. 10.1111/mec.14507 29411448

[B60] Price M. N. Dehal P. S. Arkin A. P. (2010). FastTree 2–approximately maximum-likelihood trees for large alignments. *PLoS One* 5:e9490. 10.1371/journal.pone.0009490 20224823 PMC2835736

[B61] Puschendorf R. Bolaños F. Chaves G. (2006). The amphibian chytrid fungus along an altitudinal transect before the first reported declines in Costa Rica. *Biol. Conserv.* 132 136–142. 10.1016/j.biocon.2006.03.010

[B62] Puschendorf R. Carnaval A. C. VanDerWal J. Zumbado-Ulate H. Chaves G. Bolaños F. (2009). Distribution models for the amphibian chytrid *Batrachochytrium dendrobatidis* in Costa Rica: proposing climatic refuges as a conservation tool. *Divers. Distrib.* 15 401–408. 10.1111/j.1472-4642.2008.00548.x

[B63] R Core Team (2018). *R: A Language and Environment for Statistical Computing. R Foundation for Statistical Computing.* Available at: https://www.R-project.org/ (accessed December 22, 2018).

[B64] Rebollar E. A. Antwis R. E. Becker M. H. Belden L. K. Bletz M. C. Brucker R. M. (2016a). Using “Omics” and integrated multi-omics approaches to guide probiotic selection to mitigate chytridiomycosis and other emerging infectious diseases. *Front. Microbiol.* 7:68. 10.3389/fmicb.2016.00068 26870025 PMC4735675

[B65] Rebollar E. A. Hughey M. C. Medina D. Harris R. N. Ibáñez R. Belden L. K. (2016b). Skin bacterial diversity of Panamanian frogs is associated with host susceptibility and presence of *Batrachochytrium dendrobatidis*. *ISME J.* 10 1682–1695. 10.1038/ismej.2015.234 26744810 PMC4918441

[B66] Rebollar E. A. Simonetti S. J. Shoemaker W. R. Harris R. N. (2016c). Direct and indirect horizontal transmission of the antifungal probiotic bacterium *Janthinobacterium lividum* on Green frog (*Lithobates clamitans*) tadpoles. *Appl. Environ. Microbiol.* 82 2457–2466. 10.1128/AEM.04147-15 26873311 PMC4959476

[B67] Rebollar E. A. Bridges T. Hughey M. C. Medina D. Belden L. K. Harris R. N. (2019). Integrating the role of antifungal bacteria into skin symbiotic communities of three Neotropical frog species. *ISME J.* 13 1763–1775. 10.1038/s41396-019-0388-x 30867545 PMC6776000

[B68] Reese A. T. Dunn R. R. (2018). Drivers of microbiome biodiversity: a review of general rules. Feces, and Ignorance. *mBio* 9:e1294-e18. 10.1128/mBio.01294-1218 30065092 PMC6069118

[B69] Rollins-Smith L. A. (2009). The role of amphibian antimicrobial peptides in protection of amphibians from pathogens linked to global amphibian declines. *Biochim. Biophys. Acta* 1788 1593–1599. 10.1016/j.bbamem.2009.03.008 19327341

[B70] Ryan M. J. Lips K. R. Eichholz M. W. (2008). Decline and extirpation of an endangered panamanian stream frog population (*Craugastor punctariolus*) due to an outbreak of chytridiomycosis. *Biol. Cons.* 141 1636–1647. 10.1016/j.biocon.2008.04.014

[B71] Savage J. M. (2002). *The Amphibians and Reptiles of Costa Rica: a Herpetofauna Between two Continents, Between two Seas.* Chicago: The University of Chicago Press.

[B72] Shannon C. E. (1948). The mathematical theory of communication. *BST J.* 27 379–423. 10.1002/j.1538-7305.1948.tb01338.x 30854411

[B73] Skerratt L. F. Berger L. Speare R. Cashins S. McDonald K. R. Phillott A. D. (2007). Spread of chytridiomycosis has caused the rapid global decline and extinction of frogs. *Eco. Health* 4 125–134. 10.1007/s10393-007-0093-95

[B74] Solís F. Ibáñez R. Pounds A. Bolaños F. Chaves G. Lips K. (2010). Isthmohyla rivularis. In IUCN 2011. IUCN Red List of Threatened Species. Version 2011.1. Available at: www.iucnredlist.org (accessed February 13, 2019).

[B75] Spellerberg I. F. Fedor P. J. (2003). A tribute to Claude Shannon (1916–2001) and a plea for more rigorous use of species richness, species diversity and the “Shannon-Wiener index”. *Glob. Ecol. Biogeogr.* 12 177–179. 10.1046/j.1466-822X.2003.00015.x

[B76] Šrut M. Menke S. Höckner M. Sommer S. (2019). Earthworms and cadmium – Heavy metal resistant gut bacteria as indicators for heavy metal pollution in soils? *Ecotoxicol. Environ. Saf.* 171 843–853. 10.1016/j.ecoenv.2018.12.102 30660978

[B77] Voyles J. Young S. Berger L. Campbell C. Voyles W. F. Dinudom A. (2009). Pathogenesis of chytridiomycosis, a cause of catastrophic amphibian declines. *Science* 326 582–585. 10.1126/science.1176765 19900897

[B79] Walke J. B. Becker M. H. Loftus S. C. House L. L. Teotonio T. L. Minbiole K. P. C. (2015). Community structure and function of amphibian skin microbes: an experiment with bullfrogs exposed to a chytrid fungus. *PLoS one* 10:e0139848. 10.1371/journal.pone.0139848 26445500 PMC4596541

[B80] Walke J. B. Harris R. N. Reinert L. K. Rollins-Smith L. A. Woodhams D. C. (2011). Social immunity in amphibians: evidence for vertical transmission of innate defenses. *Biotropica* 43 396–400. 10.1111/j.1744-7429.2011.00787.x

[B81] Wells K. (2007). *The Ecology and Behavior of Amphibians.* Chicago: The University of Chicago Press.

[B82] Wexler A. G. Goodman A. L. (2017). An insider’s perspective: *Bacteroides* as a window into the microbiome. *Nat. Microbiol.* 2:17026. 10.1038/nmicrobiol.2017.26 28440278 PMC5679392

[B83] Wheelwright N. T. (2000). “Conservation biology,” in *Monteverde ecology and conservation of a tropical cloud forest*, eds Nadkarni N. Wheelwright N. (New York, NY: Oxford Univ. Press), 419–456.

[B84] Whitfield S. M. Alvarado G. Abarca J. Zumbado H. Zuñiga I. Wainwright M. (2017). Differential patterns of *Batrachochytrium dendrobatidis* infection in relict amphibian populations following severe disease-associated declines. *Dis. Aquat. Org.* 126 33–41. 10.3354/dao03154 28930083

[B85] Whitfield S. M. Bell K. E. Philippi T. Sasa M. Bolaños F. Chaves G. (2007). Amphibian and reptile declines over 35 years at La Selva. Costa Rica. *PNAS* 104 8352–8356. 10.1073/pnas.0611256104 17449638 PMC1895953

[B86] Whitfield S. M. Geerdes E. Chacon I. Ballestero Rodriguez E. Jimenez R. R. Donnelly M. A. (2013). Infection and co-infection by the amphibian chytrid fungus and ranavirus in wild Costa Rican frogs. *Dis. Aquat. Org.* 104 173–178. 10.3354/dao02598 23709470

[B87] Whitfield S. M. Lips K. R. Donnelly M. A. (2016). Amphibian decline and conservation in Central America. *Copeia* 104 351–379. 10.1643/CH-15-300

[B88] Wickham H. (2016). *ggplot2: Elegant Graphics for Data Analysis.* New York, NY: Springer-Verlag.

[B89] Woodhams D. C. Alford R. A. Antwis R. E. Archer H. Becker M. H. Belden L. K. (2015). Antifungal isolates database of amphibian skin-associated bacteria and function against emerging fungal pathogens. *Ecology* 96 595–595. 10.1890/14-1837.1

[B90] Woodhams D. C. Ardipradja K. Alford R. A. Marantelli G. Reinert L. K. Rollins-Smith L. A. (2007a). Resistance to chytridiomycosis varies among amphibian species and is correlated with skin peptide defenses. *Anim. Conserv.* 10 409–417. 10.1111/j.1469-1795.2007.00130.x

[B91] Woodhams D. C. Bletz M. Kueneman J. McKenzie V. (2016). Managing amphibian disease with skin microbiota. *Trends Microbiol.* 24 161–164. 10.1016/j.tim.2015.12.010 26916805

[B92] Woodhams D. C. Brandt H. Baumgartner S. Kielgast J. Küpfer E. Tobler U. (2014). Interacting symbionts and immunity in the amphibian skin mucosome predict disease risk and probiotic effectiveness. *PLoS One* 9:e96375. 10.1371/journal.pone.0096375 24789229 PMC4005770

[B93] Woodhams D. C. LaBumbard B. C. Barnhart K. L. Becker M. H. Bletz M. C. Escobar L. A. (2018). Prodigiosin, violacein, and volatile organic compounds produced by widespread cutaneous bacteria of amphibians can inhibit two *Batrachochytrium* fungal pathogens. *Microb. Ecol.* 75 1049–1062. 10.1007/s00248-017-1095-1097 29119317

[B94] Woodhams D. C. Vredenburg V. T. Simon M.-A. Billheimer D. Shakhtour B. Shyr Y. (2007b). Symbiotic bacteria contribute to innate immune defenses of the threatened mountain yellow-legged frog. *Rana muscosa*. *Biol. Conserv.* 138 390–398. 10.1016/j.biocon.2007.05.004

