# Supplementary material for: Moving Beyond the Host: Unraveling the Skin Microbiome of Endangered Costa Rican Amphibians
Source: Front Microbiol. 2019 Sep 12;10:2060. doi: 10.3389/fmicb.2019.02060 (PMC6751270; doi:10.3389/fmicb.2019.02060)
Supplement: Supplementary file 1 [file Data_Sheet_1.docx]

**Moving Beyond the Host: Unraveling the Skin Microbiome of Endangered Costa Rican Amphibians**

Randall R. Jiménez, Gilbert Alvarado, Josimar Estrella, Simone Sommer

Supplementary Material

**Supplementary Figures**

**Supplementary Figure S1**


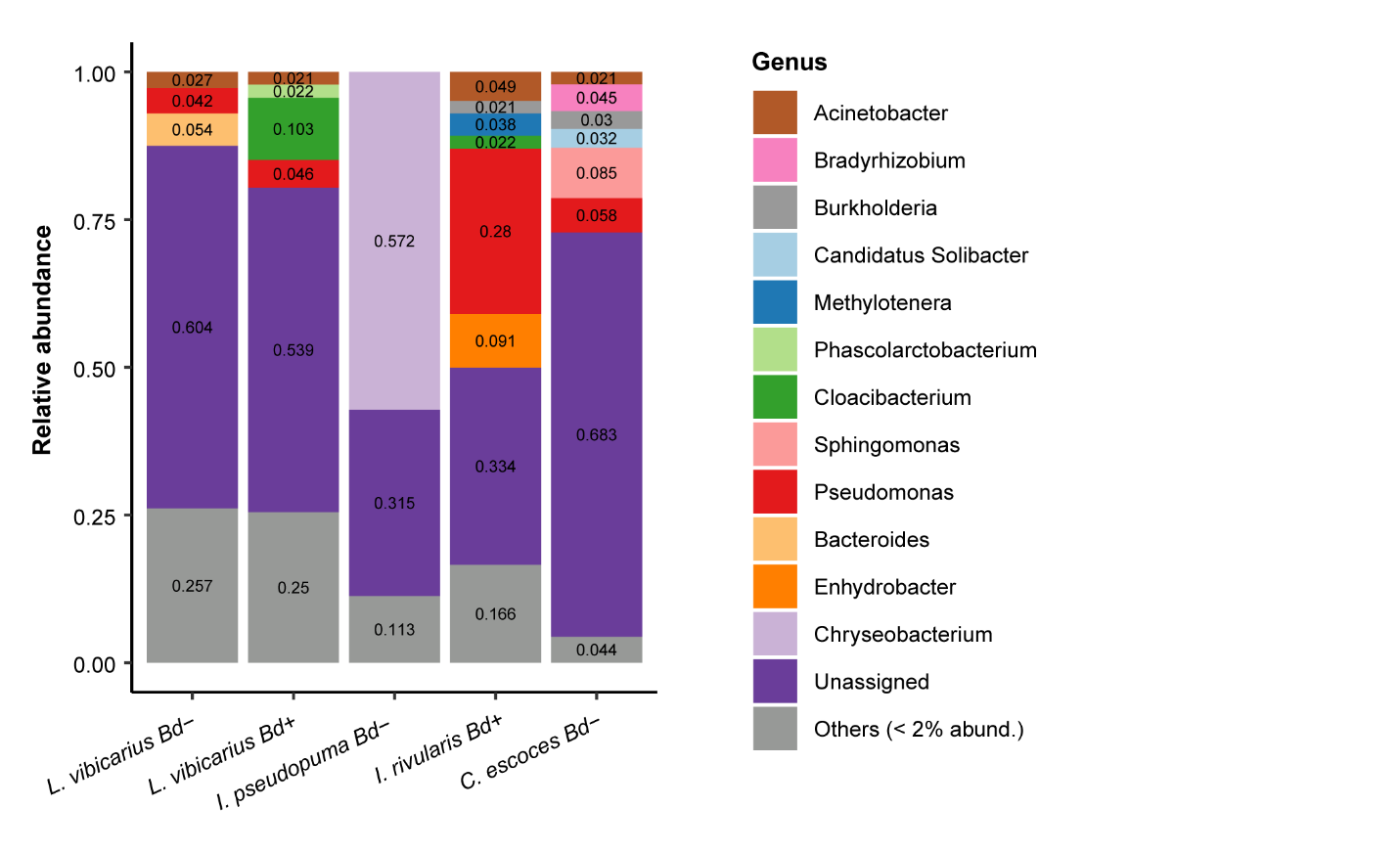
**Supplementary Figure S1**. Skin microbiome of four adult frog species grouped by *Bd*-infection status. Relative abundance of skin bacterial taxa at the genus level across species. Rare ASVs (relative abundances < 2%) were clustered together. Sample sizes are indicated in Table S1.

**Supplementary Figure S2**


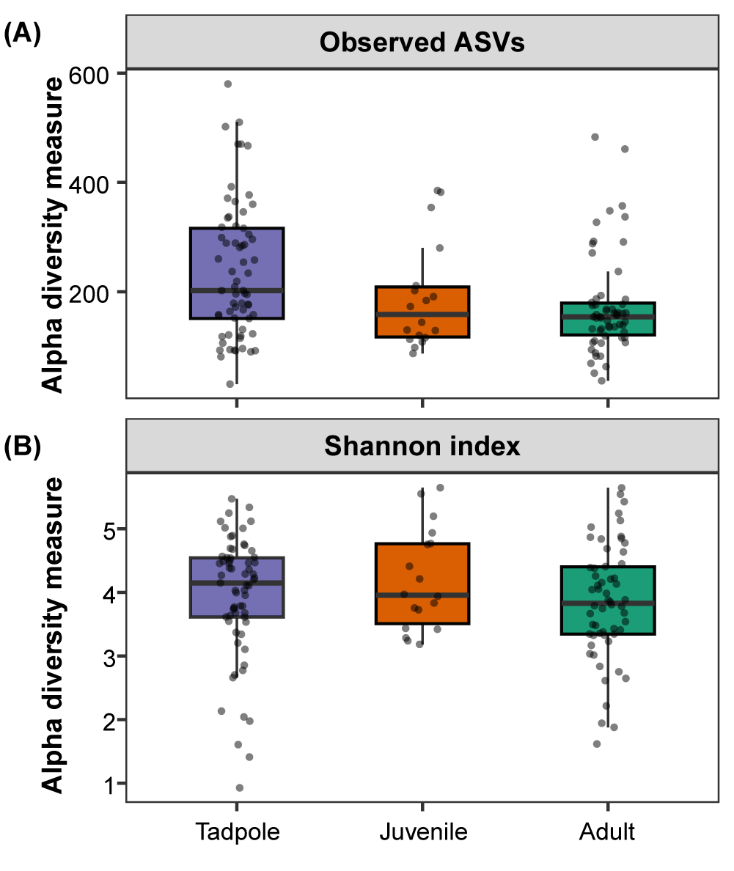


**Supplementary Figure S2.** Alpha diversity of the skin microbiome of *L. vibicarius* across life stages. **(A)** Number of observed ASVs (p < 0.001); **(B)** Shannon index in tadpoles, juveniles, and adult individuals (p = 0.95). Gray points represent an individual sample type. Sample sizes are indicated in Table S1.

**Supplementary Tables**

**Supplementary Table S1**. Amphibian samples used for skin microbiome analysis. The number of *Bd* infected animals is in parenthesis. Boxes colored in orange indicate the number of samples for the ‘species dataset’ and in green for ‘life-stage dataset’.

| Species | Site | Life stage | Year | # Samples | |
| --- | --- | --- | --- | --- | --- |
| *Isthmohyla pseudopuma* | Congo | Adult | 2016 | 3 | - |
| *Isthmohyla rivularis* | Congo | Adult | 2017 | 1(1) | - |
| *Craugastor escoces* | Congo | Adult | 2016 | 1 | - |
| *Lithobates vibicarius* | Congo | Adult | 2016 | 3 | 3 |
|  |  |  | 2017 | 9 | 9 |
|  |  | Tadpole | 2016 | - | 6 |
|  | NP | Adult | 2016 | 5(2) | 3 |
|  |  |  | 2017 | 11(1) | 10 |
|  |  | Juvenile | 2015 | - | 8 |
|  |  |  | 2017 | - | 8 |
|  |  | Tadpole | 2016 | - | 9 |
|  |  |  | 2017 | - | 10 |
|  | Lagunillas | Adult | 2015 | 13(1) | 12 |
|  |  |  | 2016 | 6 | 6 |
|  |  |  | 2017 | 8 | 8 |
|  |  | Juvenile | 2015 | - | 2 |
|  |  | Tadpole | 2015 | - | 13 |
|  |  |  | 2017 | - | 7 |
|  | Monjes | Adult | 2015 | 1 | 1 |
|  |  |  | 2016 | 6 | 6 |
|  |  |  | 2017 | 2 | 2 |
|  |  | Tadpole | 2015 | - | 8 |
|  |  |  | 2017 | - | 12 |

**Supplementary Table S2.** Putative anti-*Bd* ASVs present on the skin microbiome of four adult frog species in relation to their *Bd*-infection status.

**Supplementary Table S3.** Putative anti-*Bd* ASVs present on the skin microbiome across the life stages of *Lithobates vibicarius.*
